# The use of social simulation modelling to understand adherence to diabetic retinopathy screening programs

**DOI:** 10.1038/s41598-024-55517-4

**Published:** 2024-02-29

**Authors:** Andreia Penso Pereira, João Macedo, Ana Afonso, Raul M. S. Laureano, Fernando Buarque de Lima Neto

**Affiliations:** 1 Information Sciences, Technologies and Architecture Research Center (ISTAR-IUL), Instituto Universitário de Lisboa (ISCTE-IUL), Av. das Forças Armadas, 1649-026 Lisboa, Portugal; 2 Escola Politécnica, Computer Engineering, (POLI/EComp), Universidade de Pernambuco (UPE), Recife, 50720-001 Brazil; 3 Global Health and Tropical Medicine, GHTM, Associate Laboratory in Translation and Innovation Towards Global Health, LA-REAL, Instituto de Higiene e Medicina Tropical, IHMT, Universidade NOVA de Lisboa, UNL, Rua da Junqueira 100, 1349-008 Lisboa, Portugal; 4 Business Research Unit (BRU-IUL), Instituto Universitário de Lisboa (ISCTE-IUL), Av. das Forças Armadas, 1649-026 Lisboa, Portugal; 5 Escola Politécnica, Computer Engineering (POLI/PPG-EC), Universidade de Pernambuco (UPE), Rua Benfica, 455-Bloco ‘C’, Recife, 50720-001 Brazil

**Keywords:** Computational simulation, Agent-based models, Logistic regression, Fuzzy logic, Diabetic retinopathy, Screening adherence rate, Disease prevention, Preventive medicine, Health care, Public health, Population screening

## Abstract

The success of screening programs depends to a large extent on the adherence of the target population, so it is therefore of fundamental importance to develop computer simulation models that make it possible to understand the factors that correlate with this adherence, as well as to identify population groups with low adherence to define public health strategies that promote behavioral change. Our aim is to demonstrate that it is possible to simulate screening adherence behavior using computer simulations. Three versions of an agent-based model are presented using different methods to determine the agent’s individual decision to adhere to screening: (a) logistic regression; (b) fuzzy logic components and (c) a combination of the previous. All versions were based on real data from 271,867 calls for diabetic retinopathy screening. The results obtained are statistically very close to the real ones, which allows us to conclude that despite having a high degree of abstraction from the real data, the simulations are very valid and useful as a tool to support decisions in health planning, while evaluating multiple scenarios and accounting for emergent behavior.

## Introduction

Diabetic retinopathy (DR), ICD-9 code 362.0, is a complication of diabetes that causes structural changes in the blood vessels of the retina. It is currently one of the main causes of blindness in developed countries^1^. As DR is asymptomatic until the later stages, patients with diabetes should have regular eye tests^1,2^. Several countries have therefore implemented population-based DR screenings^3^.

The literature demonstrates that the success of screening programs depends to a large extent on the adherence of the target population, but as far as we know there is a gap in the study of the behavioral mechanisms behind the phenomenon (as demonstrated in “Literature review”).

In order to bridge this gap, our research focuses on the development of computer simulation models that make it possible to understand the factors that correlate with adherence rates, identify population groups with particularly low adherence and may help to support decisions in health planning, while evaluating multiple scenarios and accounting for emergent behavior.

In this article we are mostly focused on the first step of the process: how to predict the rate of adherence to population-based screenings through computational simulation models with a high level of abstraction. More specifically, this article aims to (i) demonstrate that it is possible to develop a computational simulation model that faithfully portrays the individual decision to adhere to screening or not, using the intrinsic features of the diabetic patients and of the screening programs, (ii) demonstrate that a simulation model with the aforementioned characteristics can be used in contexts other than the one in which the data for its development was collected, (iii) hopefully, demonstrate the utility of combining ABM and fuzzy logic in models that intend to simulate human behavior. To this end, we developed three versions of an agent-based simulation model (ABM), using a logistic regression equation and fuzzy logic components to predict the individual decision concerning whether to adhere to the screening or not. By integrating the fuzzy components with the result of the logistic linear regression, with parametrizable weights, the proposed model can be used to compare the two methods, and a combination of both techniques.

The first set of simulations, in which the individual decision on whether to adhere to the screening or not was based on logistic regression, proved to be good at replicating reality and useful in staging scenarios in a specific geographic context, but we recognize that its scalability and level of abstraction is limited, as it is mainly based on behaviors previously observed in a concrete screening program^27^. The second set of simulations allows us to overcome the aforementioned limitations, despite some decline in the accuracy of the previsions, by replacing the logistic regression equation with three fuzzy logic components to simulate individual decision-making. Finally, a third set of simulations was performed, combining the logistic regression and fuzzy components in equal proportions.

## Literature review

The first computer simulations of DR screenings date back to the 1990s and mainly used Markov chains to demonstrate the cost-effectiveness of implementing population-based screening programs^4–7^. In subsequent years, and with a broad consensus on the cost-effectiveness of population-based DR screenings, researchers started to focus on the analysis of different screening alternatives. Several simulation models were developed to compare screening methods^8–12^, the results of adopting different screening intervals^9–17^, and to analyze the cost-effectiveness of telemedicine^18–23^. We highlight the works of Davies and his colleagues, who developed a simulation model based on discrete events (DES) to stage different screening intervals^9–12^ and found that the population's adherence to the screening plays a decisive role in its success. However, in subsequent models the authors continued to adopt a fixed probability of adherence and no attempt was made to model the subjects' individual behavior^9–12^. An attempt to include human behavior was made by Schmidt and Brailsford^24^, by incorporating the Health Belief Model (HBM) into a DES model that produces a result (behavior—output) through a combination of several factors (inputs) that influence screening adherence. In this model each patient is an individual entity, with their own features. This approach was implemented using numerical attributes to represent the various features of the diabetics (number of times they adhered to previous screenings, perception of general health status, current DR status, information and anxiety regarding DR, and educational qualifications). The probability of participating in the screening was calculated as a binary variable and the model uses only artificial data, leading to results that are theoretical artifacts which lack validation with real data. This research also stresses the difficulty of incorporating qualitative variables, such as those used by the HBM, in DES models, emphasizing the need for the use of another type of technique^24^. Supplementary Table S1 provides further details on the strengths and limitations of each of these studies.

The literature suggests that ABM are a good alternative for the study of systems in which individual behavior has a relevant impact, since they are composed of networks and processes formed by interactive and adaptive agents^26^. In fact, in an ABM the social system is represented as a set of autonomous agents capable of taking decisions. In each iteration, each agent individually assesses their situation and makes decisions based on a set of rules, then takes a certain action. Even a simple ABM can exhibit complex behavior patterns and provide valuable information on the dynamics of the real-world system it simulates^25,26^. Among the main advantages of using such an approach, we highlight its ability to simulate different scenarios and emergent behavior that is not explained by classic theories, such as the adoption of behaviors that repeatedly do not result in the best outcome; heterogeneity and interactions between agents; flexibility and the possibility of following the evolution of a system^26^.

The concept of fuzzy logic, introduced by Lotfi Zadeh in 1965, is based on the observation that human beings make decisions based on imprecise, subjective and non-numerical information^28,29^. Thus, fuzzy sets are mathematical entities that aim to represent imprecise information and give models the ability to recognize, represent, manipulate, interpret, and use vague and/or subjective information, allowing for a high level of abstraction in relation to the original data^29^. Techniques based on fuzzy logic are therefore especially suitable for simulating human behavior, having already been used quite successfully for this purpose^30^, and fuzzy rules can be embedded in within the intelligent agents of the ABM^31^.

## Methods

**Figure 1:**
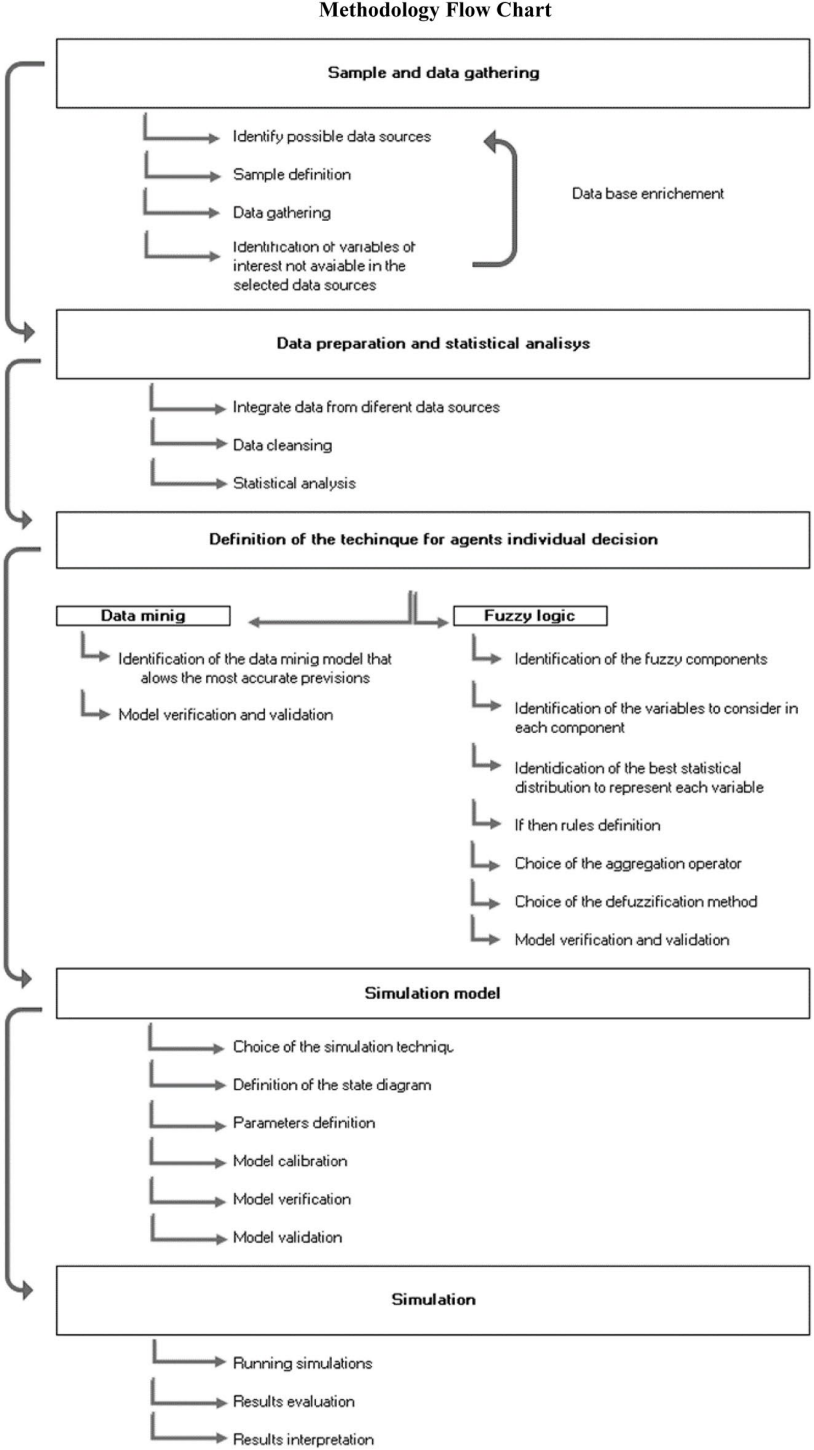
Methodology flow chart.

This research focuses on the concept of model development driven by real data^32^. Thus, the research process began with the important steps of identifying sources and collecting, integrating, and processing data. After this, the modelling process itself began. The following flowchart (Fig. 1) illustrates the main steps performed, which will be described in greater detail in the following subsections.

### Sample and data gathering

**Figure 2:**
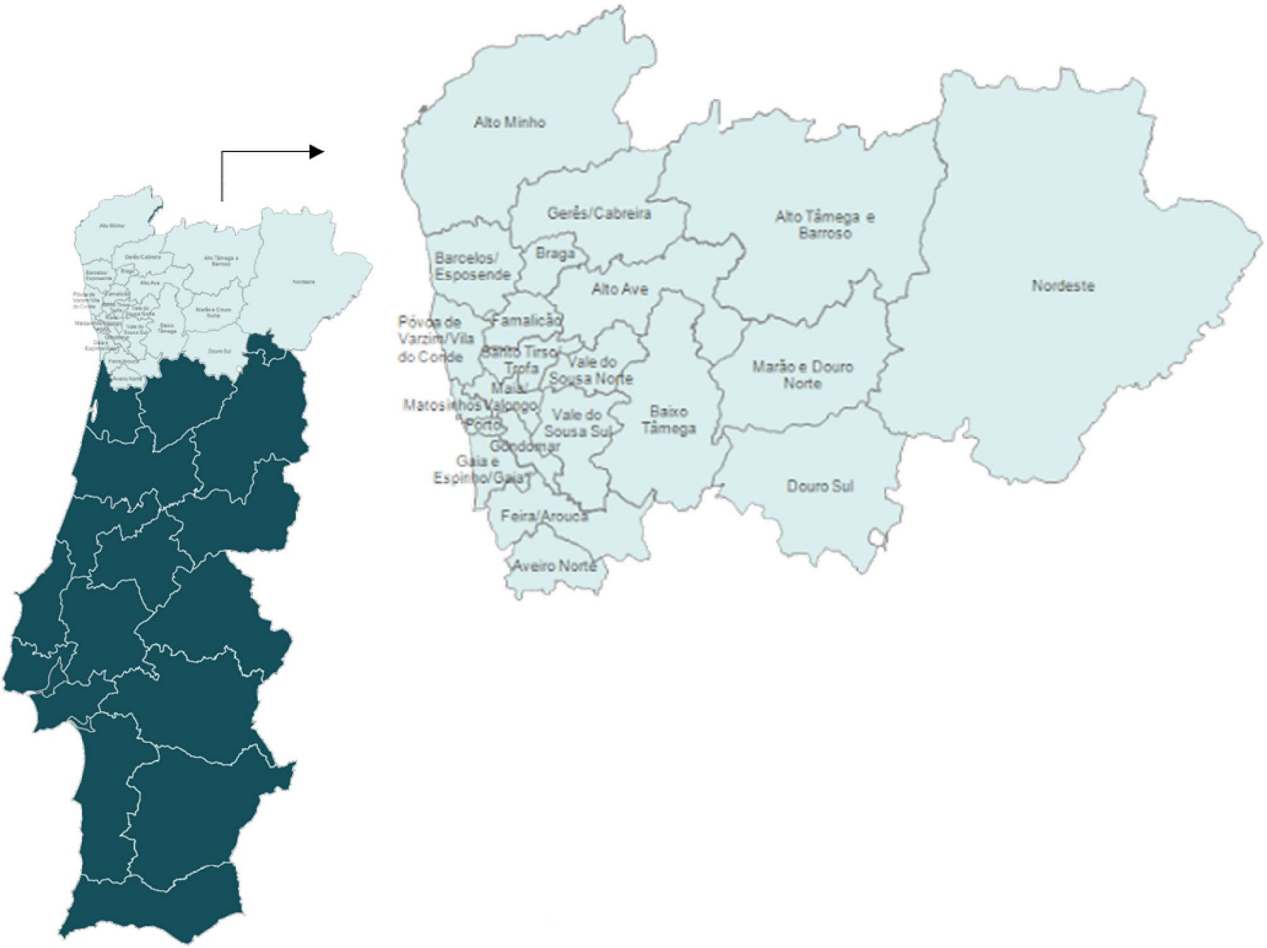
ARSN location and geographical coverage.

This research used data on all calls for DR screening between the years 2013 and 2018, provided by the Portuguese Northern Region Health Administration (ARSN). Figure 2 illustrates the geographic area covered by ARSN.

The sample consists of 271,867 calls for DR screening, which corresponds to 108,620 different diabetics. The following variables were used: age; gender; professional status; existence of telephone contact for sending reminders; Health Centre Cluster (ACES); Primary Health Care Unit; type of Primary Health Unit; existence of a family doctor; reason for exemption from payment of charges for services, when applicable; number of consultations at the Primary Care Unit in the last 12 months; type of diabetes (I or II); Body Mass Index (BMI); blood glucose levels (HBA1C); month of call for screening; days elapsed between calls; number of times the diabetic was called; last screening result; percentage of times the diabetic adhered to previous screenings. Subsequently, data from the National Institute of Statistics (INE) was used to obtain the variables "income (median)" and "educational qualifications (distribution by postal code with 7 digits)”^33^, as these variables are identified in the literature as closely related to the adherence rate^24^. For the classification of geographical areas according to the degree of urbanization, data from the Typology of Urban Areas 2014 (TIPAU, 2014), available on the INE website^33^, was used.

All methods of data gathering were carried out in accordance with relevant guidelines and regulations.

The authors did not have any direct contact with the subjects participating in the study.

The data obtained from INE are publicly available and of a general nature, not allowing the identification of the subjects involved^33^.

The data provided by ARSN were collected by the institution, in accordance with the legislation applicable in the Portuguese Public Administration, including informed consent from all subjects and/or their legal guardian(s).

Moreover, the data provided by ARSN for this research went through a set of mechanisms that guarantee the protection of privacy (for example encryption and anonymization), and all the procedures were duly endorsed by the ARSN ethics committee, in strict compliance with all issues related to access to Public Administration data, and the data protection regime.

The present research does not include the use of experimental protocols.

### Data preparation and Statistical analysis

A large percentage of the work in data analysis involves preparing the data^32^. Hence, in this phase it was necessary to integrate data from different data sources and perform data cleansing: identifying impossible or incorrect values for specific variables, cases that should not be in the study (because they do not meet the inclusion criteria), duplicate cases, missing data, and outliers, while also ensuring that the same value for string variables is always written in a coherent manner throughout the data set. The SPSS Modeler 18.2 software^34^ was used to carry out this step.

In a second phase, a descriptive statistical analysis was carried out, aiming to identify the variables that best explain adherence to screening. The results of the statistical analysis are presented in “Sample and data gathering”.

### The diabetic’s individual decision

**Figure 3:**
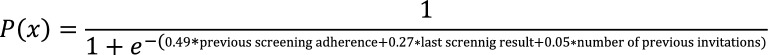
Logistic regression equation.

In order to select the data mining model, eight models were tested using SPSS Modeler 18.2 software^34^: decision trees (C5, Tree-AS, CHAID, Quest, C&R Tree), neural network, Bayesian network and logistic regression. For this study, the adherence to screening variable was used as a dichotomous dependent variable. A set X of 21 independent variables was considered. The logistic regression model (Fig. 3) revealed the most accurate results (62.23% correct in the training set, AUC = 0.68 and 63.62% in the testing set, AUC 0.681). Only 3 independent variables were included in the regression model that was generated, since the others were discarded (using the stepwise method) due to their low significance in the model. The predictors of the behavior of adherence to the screening are the percentage of times the diabetic had previously adhered to the screening, the last screening result, and the number of times the diabetic was called for DR screening, which is in line with the literature^24^.

As an alternative to the data mining model, a set of fuzzy components was developed to measure the result of the individual decision on whether to adhere to the screening or not.

The fuzzy components, as well as the variables that constitute each component, were established on the basis of the statistical analysis results, those in the literature that focus on explaining the rate of adherence to health programs, and the HBM^23,24^. This analysis resulted in three common-sense fuzzy components: “access barriers”, “knowledge of the disease”, “quality/strategy of the screening program”. The selection of the representative function for the variables that comprise each component was based on an analysis of the distribution of real data. The “access barriers” component comprises variables B1, B2, B3 and B4. B1 concerns the perception of access barriers due to age. “Difficult access due to age” is defined by the linear function that passes through the points [0, 1], [100, 0]. “Easy access” is defined by the linear function that passes through the points [0, 0] and [100, 1]. Variable B2 corresponds to the perception of access barriers as a function of income. "Difficult access due to income" is defined by the normal distribution of the mean 50,000 euros/year and standard deviation 17,000 euros/year. The classification "easy access due to income" corresponds to the maximum of two normal distributions with averages of 0 and 100,000 euros/ year respectively and standard deviations of 17,000 euros/year. B3 corresponds to the perception of access barriers depending on the location of the screening. “Difficult to access due to screening location” is defined by the linear function that passes through the points [0, 0], [100, 1]. “Easy access due to screening location” is defined by the linear function that passes through the points [0, 1] and [100, 0]. B4 corresponds to the perception of access barriers depending on the degree of urbanization of the place of residence. The “difficult access due to the degree of urbanization” is defined by the normal distribution of mean 0.3 and standard deviation 0.1. The classification “easy access due to the degree of urbanization” corresponds to the maximum of two normal distributions with means 0 and 0.5 respectively and standard deviations 0.1. The component relating to knowledge about the disease comprises variables C1, C2 and C3. Variable C1 assesses knowledge of the disease as a function of age. "High knowledge level due to age" is defined by a normal distribution of the mean 65 years and standard deviation 30. "Low knowledge level due to age" corresponds to the maximum of two normal distributions with means 18 and 100 years respectively and deviation pattern 30. Variable C2 corresponds to knowledge of the disease as a function of educational qualifications. “High knowledge level due to educational qualifications” is defined by a linear function that passes through the points [0, 0] and [100, 1]. “Low knowledge level due to educational qualifications” is defined by a linear function that passes through the points [0, 1] and [100, 0]. Variable C3 corresponds to knowledge of the disease as a function of the percentage of times the agent previously adhered to screening. “High knowledge level due to prior adhesion” is defined by a linear function that passes through the points [0, 0] and [100, 1]. “Low knowledge level due to prior adhesion” is defined by a linear function that passes through the points [0, 1] and [100, 0]. The component relating to the quality of the screening strategy comprises variables E1, E2 and E3. Variable E1 corresponds to the quality of the strategy in terms of the sending of reminders. “High quality, considering sending reminders” is defined by a linear function that passes through the points [0, 0] and [100, 1]. “Low quality, considering sending reminders” is defined by a linear function that passes through the points [0, 1] and [100, 0]. Variable E2 corresponds to the quality of the strategy considering the waiting time at the time of screening (in minutes). “High quality, considering the waiting time” is defined by a linear function that passes through the points [0, 1] and [500, 0]. “Low quality, considering the waiting time” is defined by a linear function that passes through the points [0, 0] and [500, 1]. Variable E3 corresponds to the time (in weeks) between sending the call notice and the date of the screening. "High quality, considering advance notification of the call" is defined by a normal distribution of mean 4 and standard deviation 2. "Low quality, considering advance notification of the call" corresponds to the maximum of two normal distributions with means 0 and 8 respectively and standard deviations 2. Finally, a random noise was added, whose magnitude is controlled by the “variability” parameter. In each component, rules of the IF–Then type were defined, so that if the easy/high/high value is obtained in at least half of the variables that comprise it, there is a strong probability that the agent will adhere to the tracking. Therefore, for the “barriers of access” component, 16 rules were defined, 8 for the “knowledge of the disease” component and 8 for the “quality/strategy of the screening program” component, resulting in a total of 32 IF–then rules (listed in Supplementary Information S2). The maximum as aggregation operator and the Mamdani Fuzzy Inference Method were used, and the defuzzification of each component was performed by the Centre of Gravity (COG) method^28,29^. The final result corresponds to the average of the results of the three components.

### Simulation model

**Figure 4:**
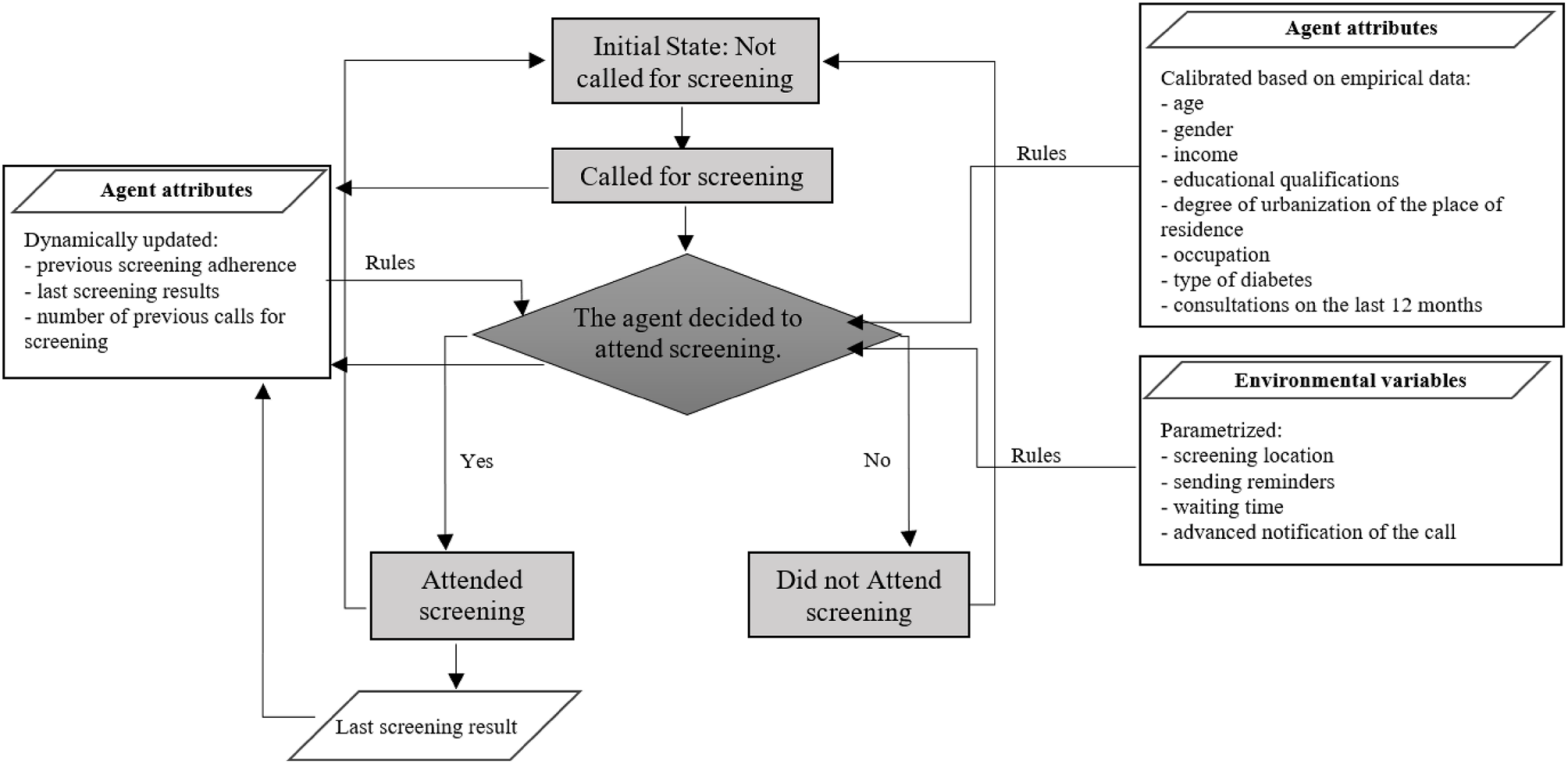
ABM Conceptual Model: Status Diagram and Decision Process.

The ABM was developed using NetLogo 6.1.1 software^35^, a simulator written in Scala language. The status diagram that was implemented (Fig. 4) contemplates four possible states: (i) not called; (ii) called; (iii) attended screening; (iv) did not attend screening. Initially, all diabetics assume the “not called” status. At the beginning of the simulation, each diabetic is called for screening by means of an invitation letter. At that moment, the diabetic assumes the status “Called for screening” (until the date of the screening). On the date of the screening, it is verified whether the diabetic has attended the screening or not. According to the diabetic’s action, he can assume the status “attended screening” or “did not attend screening”, as the case may be. After this phase, a new cycle begins in which the diabetic returns to the “Not called” state until the stipulated interval between screenings elapses. At that moment, a new invitation letter is issued, and the diabetic again assumes “Called for screening” status, repeating the entire process.

By integrating the fuzzy components with the result of the logistic linear regression, the current model allows the two methods to be compared, as well as the results obtained with the use of different weights selected by the user. The information regarding the screening strategy was based on the opinion of ARSN experts and on an analysis of official documents provided by the institution^36^. Hence, the following parameters were used: Screening location = Primary Health Care Unit; Screening test sensitivity = 96%; Screening test specificity = 94%; Probability of a positive screening test = 4%; Probability of a negative screening test = 93%; Probability of an inconclusive screening test = 3%; Screening intervals = 52 weeks.

### Simulations

A virtual population of 10,000 diabetics was generated and the call for screening was simulated over a period of ten screening cycles. A 52-week interval was defined between screenings. The initial population of agents was designed according to the characteristics of the ARSN diabetic population, and the model was initialized with the parameters measured from the available data. Five simulations were performed for each version of the model. In order to test the model's ability to capture geographic specificities, the simulation results obtained for each subregion were compared with the real adherence rates.

For the version that bases the individual decision of whether or not to adhere to the screening exclusively on fuzzy components, the data set was divided into two groups: training and testing. Data relating to the geographical areas of Tâmega e Sousa, Cávado, Douro, Trás-os-Montes and the Metropolitan Area of Porto, which corresponds to 66.41% of the total diabetic population covered by the ARSN, was used for training. Data relating to the geographical areas of Alto Minho, Ave and Entre Douro e Vouga, which correspond to 33.59% of the ARSN diabetic population, was reserved for testing. It was not possible to conduct a similar procedure for the version that only uses the logistic regression model because the model needs previous regional screening information to run.

For the version that uses fuzzy components, we also compared the results obtained for the entire population, with the ones obtained defining sets based on specific subgroups determined by a previous cluster analysis. To this end, we performed a cluster analysis using SPSS Modeler 18.2 software^34^. The initial data set of 271,867 calls for DR screening, corresponding to 108,620 different diabetics, was divided in two clusters, using the TwoStep Cluster Analysis procedure's algorithm. The model summary table indicates that two clusters were found based on seven input features. The cluster quality chart indicates that the overall model quality is "Fair". 50.9% (138,258) of the records were assigned to the first cluster and 49.1% (133,609) to the second. The cluster means suggest that the clusters are well separated for some of the variables, but to better evaluate the quality of the model, chi-squares and Cramér's V tests were performed for each variable. Although the chi-square tests point to the significance of the relation between clusters and all the input variables, this is mostly due to the large dimension of the data set. In this conditions, Cramér's V tests are better suitable to understand the correlations between input variables and clusters. The Cramer’s V tests revealed that only two of the seven variables have strong correlations with the cluster variable: age groups and occupation.

So, tendentially Cluster 1 is comprised by younger diabetics, mostly active, and Cluster 2 by older diabetics, mostly retired. The main aspects of the cluster analysis were included in the manuscript and the details are available in Supplementary Information—Tables S3 and S4.

## Results

### Statistical analysis results

The statistical analysis results (Supplementary Information Table S5) are, in general, consistent with those found in the literature on population-based screening. Younger and older diabetics tend to adhere less to screening, as well as those earning higher incomes^37^. Higher educational qualifications, as well as a regular habit of using primary health care—visits to the health unit in the last 12 months—are conducive to higher rates of adherence^37–39^. Diabetics who received a higher number of invitations for previous screening and who had adhered more frequently in the past had higher rates of adherence^24,39^. There are, however, results that are not supported by the literature. Contrary to expectations^37–39^, men in the ARSN adhere more to screening, and diabetics with previous positive results have lower adherence rates in the next screening. Regarding this second result, a scientific article focusing on the perspective of one of the main hospitals in the northern region which is an integral part of the ARSN screening program may indicate a possible explanation^40^. In fact, the lack of communication between hospital services and primary health care often results in calls for screening being sent to diabetics who are already being followed up and undergoing treatment in a hospital environment.

### Simulation results using logistic regression only

**Figure 5:**
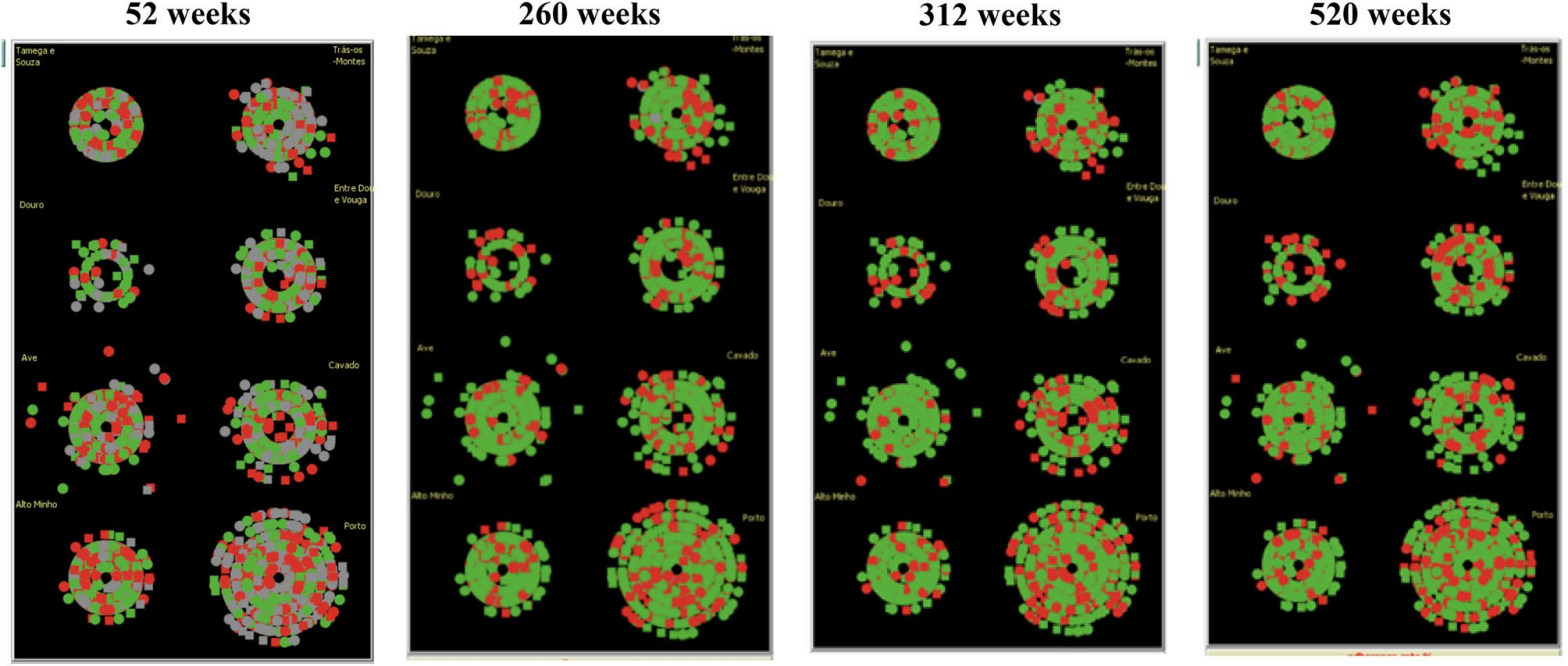
Evolution of the adherence rate in the different geographic areas throughout the simulation (output Netlogo). Each point on the diagram corresponds to a diabetic. The points cluster in concentric shapes which represent the different subregions (8) and whose size reflects the number of people with diabetes. The approximation of each point to the centre is determined by the income bracket of the diabetic represented. The colour assigned to each point corresponds to the status of the diabetic: grey indicates not called or called for screening; green indicates attended screening; red indicates did not attend.

The objective of this first set of simulations was to compare the simulated adherence rate with the real ARSN adherence rate, using logistic regression only in the agent decision process. In order to test the model's ability to capture geographic specificities, the simulation results obtained for each subregion were compared with the real adherence rates. The model was run for 520 simulated weeks to ensure convergence of results. In all cases, there was a significant initial increase in the global adherence rate, after which the model converges to an average adherence rate of 67.6%, with a standard deviation of 0.16%. The real adherence rate is slightly lower (66.6%). Figure 5 illustrates the simulation results obtained after 52, 260, 312 and 520 weeks.

When the model stabilizes, the simulated values approach the real ones. Only in one subregion (Douro) does the actual value of the adherence rate fall outside the 99% confidence interval. The simulation results also reflect the geographical asymmetries well (Table 1, Fig. 7).

**Table 1:** Simulation results versus real data.

	NUTS II	Simulation results		Real results (%)	Differences (%)	Confidence interval 95%	Confidence interval 99%
		Mean (%)	Standard deviation (%)				
	Agent decision based on logistic regression						
	Alto Minho	74.30	0.81	74.10	0.20	]72.99; 75.01[	]72.33; 75.67[
	Ave	72.44	0.67	72.09	0.35	**]71.17; 72.83[**	]70.62; 73.38[
	Cávado	66.75	0.83	67.02	−0.27	]64.97; 67.03[	]64.29; 67.71[
	Douro	68.13	0.29	66.86	1.27	**]67.64; 68.36[**	**]67.40; 68.60[**
	Entre Douro e Vouga	66.95	0.63	66.25	0.70	]65.22; 66.78[	]64.70; 67.30[
	Metrop. Area of Porto	65.35	1.01	63.38	1.97	**]63.75; 66.25[**	]62.92; 67.08[
	Tâmega e Sousa	69.50	0.76	67.59	1.91	**]68.06; 69.94[**	]67.44; 70.56[
	Trás-os-Montes	66.83	0.65	65.69	1.14	]63.58; 66.42[	]62.65; 67.35[
	Agent decision based on fuzzy components						
Training	Cávado	65.58	1.42	67.02	−1.44	]63.82; 67.34[	]62.66; 68.50[
	Douro	65.44	0.66	66.86	−1.42	**]64.18; 65.82[**	**]63.64; 66.36[**
	Metrop. Area of Porto	62.00	1.00	63.38	−1.38	**]60.76; 63.24[**	]59.94; 64.06[
	Tâmega e Sousa	67.03	1.13	67.59	−0.56	]65.60; 68.40[	]64.67; 69.33[
	Trás-os-Montes	62.79	0.90	65.69	−2.90	**]60.88; 63.12[**	**]60.15; 63.85[**
Test	Alto Minho	70.92	0.81	74.10	−3.18	**]68.99; 71.01[**	**]68.33; 71.67[**
	Ave	70.78	0.87	72.09	−1.31	**]68.92; 71.08[**	**]68.21; 71.79[**
	Entre Douro e Vouga	65.40	0.93	66.25	−0.85	**]63.85; 66.15[**	]63.09; 66.91[
	Agent decision based on fuzzy components—Cluster 1						
Training	Cávado	64.62	1.48	65.46	−0.84	]63.32; 65.91[	]62.92; 66.32[
	Douro	66.25	0.84	68.72	−2.47	**]65.51; 66.98[**	**]65.28; 67.21[**
	Metrop. Area of Porto	61.70	1.30	62.34	−0.64	]60.56; 62.84[	]60.20; 63.20[
	Tâmega e Sousa	66.48	1.09	67.41	−0.93	]65.52; 67.43[	]65.22; 67.73[
	Trás-os-Montes	61.42	1.50	65.22	−3.80	**]60.11; 62.73[**	**]59.70; 63.15[**
Test	Alto Minho	70.80	0.78	72.34	−1.54	**]70.12; 71.49[**	**]69.90; 71.7[**
	Ave	69.65	0.78	70.28	−0.63	]68.96; 70.33[	]68.75; 70.55[
	Entre Douro e Vouga	64.33	1.59	65.10	−0.77	]62.93; 65.73[	]62.49; 66.17[
	Agent decision based on fuzzy components—Cluster 2						
Training	Cávado	67.93	0.70	68.83	−0.90	**]67.31; 68.54[**	**]67.12; 68.73[**
	Douro	66.61	0.61	64.66	1.95	**]66.07; 67.14[**	**]65.90; 67.31[**
	Metrop. Area of Porto	64.27	0.74	64.33	−0.06	]63.62; 64.92[	]63.41; 65.13[
	Tâmega e Sousa	67.89	0.59	67.76	0.13	]67.37; 68.40[	]67.21; 68.57[
	Trás-os-Montes	64.65	0.56	66.37	−1.72	**]64.16; 65.15[**	**]64.01; 65.30[**
Test	Alto Minho	71.47	0.44	76.04	−4.57	**]71.08; 71.85[**	**]70.96; 71.97[**
	Ave	71.91	0.50	73.74	−1.83	**]71.47; 72.36[**	**]71.33; 72.49[**
	Entre Douro e Vouga	66.90	0.38	67.32	−0.42	**]66.57; 67.24[**	]66.46; 67.34[
	Agent decision based on a combination of logistic regression and fuzzy components						
Training	Cávado	65.76	1.23	67.02	−1.26	**]63.47; 66.53[**	]62.47; 67.53[
	Douro	67.13	0.56	66.86	0.27	]66.30; 67.70[	]65.85; 68.15[
	Metrop. Area of Porto	61.44	1.13	63.38	−1.94	**]59.60; 62.40[**	**]58.67; 63.33[**
	Tâmega e Sousa	64.67	0.98	67.59	−2.92	**]62.78; 65.22[**	**]61.98; 66.02[**
	Trás-os-Montes	61.56	0.87	65.69	−4.13	**]59.92; 62.08[**	**]59.21; 62.79[**
Test	Alto Minho	71.42	0.79	74.10	−2.68	**]70.02; 71.98[**	**]69.37; 72.63[**
	Ave	71.30	0.86	72.09	−0.79	**]69.93; 72.07[**	]69.23; 72.77[
	Entre Douro e Vouga	68.21	0.83	66.25	1.96	**]66.97; 69.03[**	**]66.29; 69.71[**

Bold cells signal cases where the actual value of the adherence rate falls outside the confidence interval.

### Simulation results using fuzzy components only

**Figure 6:**
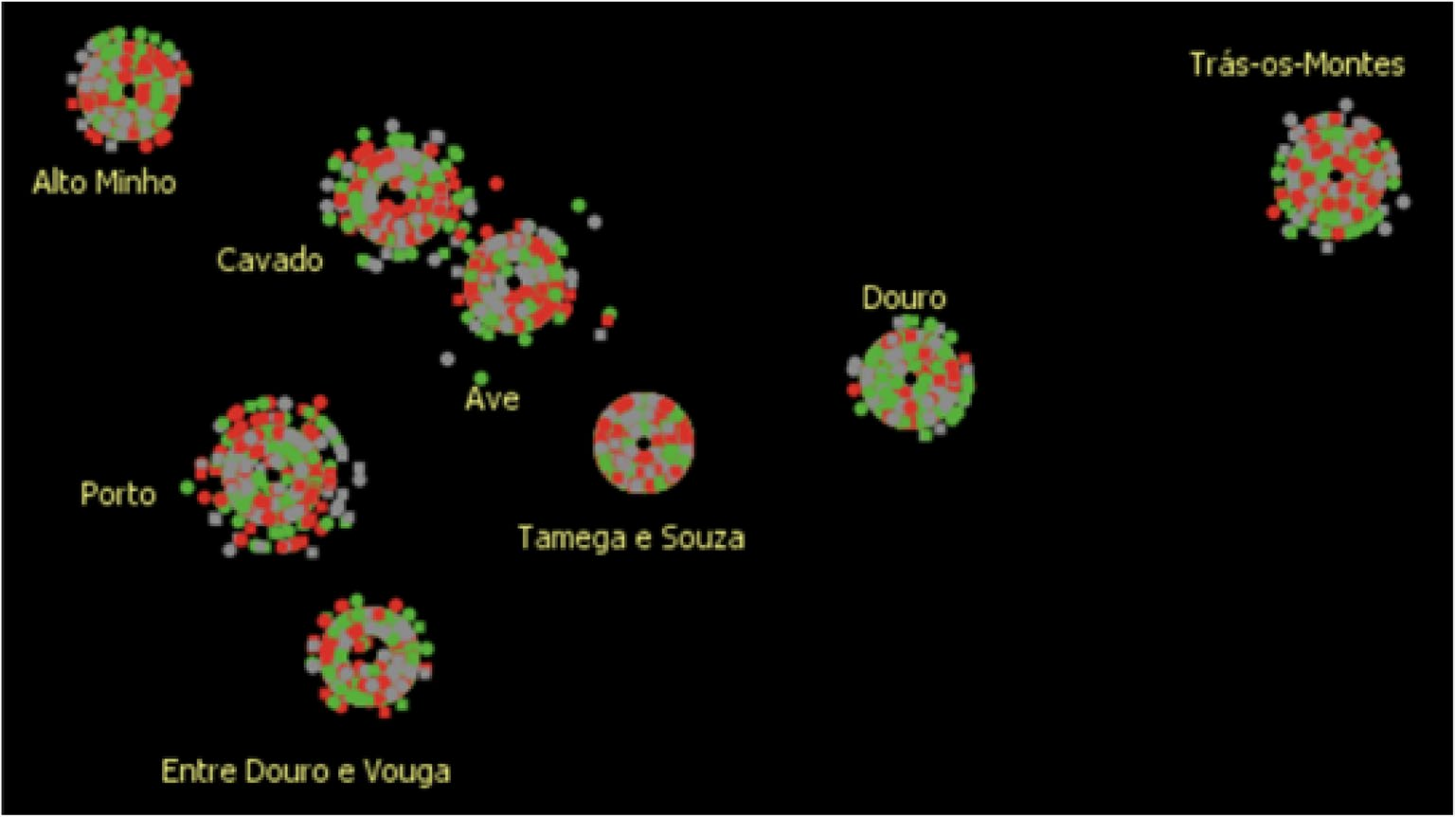
Adherence rate by geographic area (output Netlogo). Each point in the diagram corresponds to a diabetic residing in a certain health subregion at a certain moment in the simulation. The points cluster in concentric shapes which represent the different subregions (8) and whose size reflects the number of people with diabetes. The approximation of each point to the centre is determined by the income bracket of the diabetic represented. The colour assigned to each point corresponds to the status of the diabetic: grey indicates not called or called for screening; green indicates attended screening; red indicates did not attend.

In this second phase, the simulations were obtained using fuzzy components only to establish the agent decision rules. The initial population of agents was designed according to the characteristics of the diabetic population in each of the geographic areas that belong to the ARSN. Since all the real data belongs to the same Regional Health Administration, the screening strategy is similar in all sub-regions (both for training and testing). Hence, sending reminders is still a very incipient practice and the screening is carried out in primary care units in all the eight subregions under analysis. It has not yet been possible to obtain information on the other variables that comprise the “quality/screening strategy” component. Therefore, it was assumed that the call is sent 4 weeks in advance in all locations and that the waiting time on the screening day is always 10 min. Five simulations were performed. Figure 6 corresponds to Netlogo's graphic output obtained with the first simulation performed.

**Figure 7:**
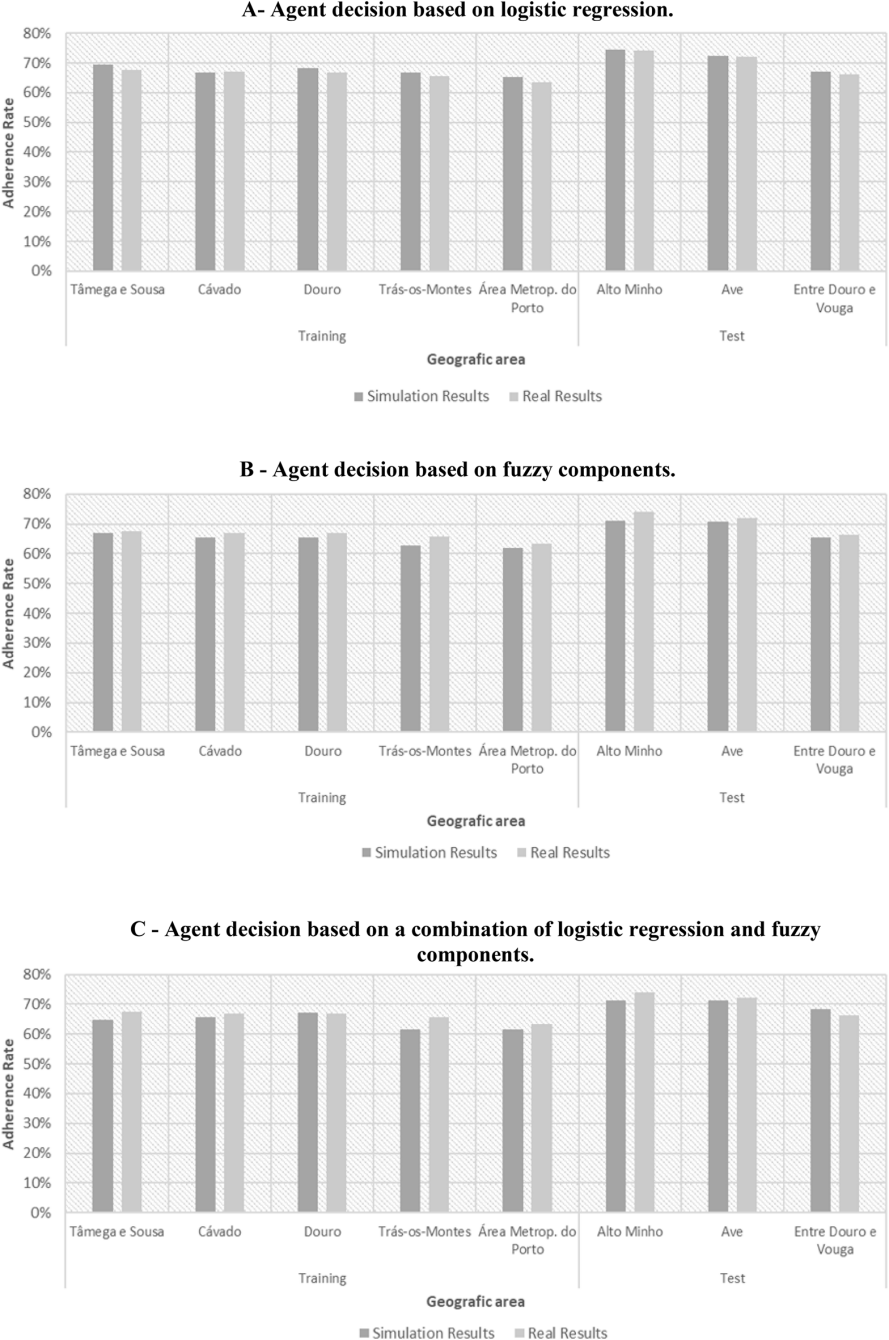
Simulation results versus real data.

Table 1 and Fig. 7 summarize the results obtained in comparison to the real data.

The overall ARSN adherence rate obtained using the simulation model is 1.55% below the region's real adherence rate (65.05% versus 66.6%). In fact, the results obtained with the simulations are slightly below the actual adhesion rate in all geographic subregions, with the smallest difference being registered in Tâmega and Sousa (0.56%) and the largest in Alto Minho (3.18%). In four subregions the actual value of the adherence rate does not belong to the 99% confidence interval. However, the model is able to effectively capture the nuances between different regions in terms of adherence to screening.

The results obtained using the previous defined clusters are very satisfactory, and, particularly for cluster 1, the simulation results are in fact a better representation of reality, when compared with the results obtain using the entire population (Table1).

It should also be noted that the adjustment to reality in the test set and the model’s ability to predict higher adherence rates supports the belief that the model has a predictive capacity in new contexts.

### Simulation results using a combination of logistic regression and fuzzy components

In this last set of simulations, the agent decision results from a combination of the results obtained with logistic regression and with fuzzy components, in a ratio of 50/50. The simulations were performed as described in the two previous sections. Table 1 and Fig. 7 illustrate the results obtained.

The overall ARSN adherence rate obtained was 1.52% below the region's real adherence rate (65.08% versus 66.6%). The biggest difference (absolute value) was registered in Trás-os-Montes (4.13%) and the smallest in Douro (-0.27%). In five subregions the actual value of the adherence rate does not belong to the 99% confidence interval.

### Comparation of the results obtain with the three versions of the ABM

The results obtained are close to the real ones, even though four of the eight subregions in the version that uses fuzzy components present real values that fall outside the 99% confidence interval for the mean of the simulation results. Therefore, the model captures the geographic asymmetries very well. The use of the fuzzy components leads to a high level of abstraction from the real data and shows predictive capability in new contexts (the test set), which attests to the validity of the model for the study of this problem and its usefulness as a predictive tool for public health planning. In fact, the use of logistic regression (version 1) led to the best global result: a predicted adherence rate of 67.6%, a difference of only 1% in relation to the real value (66.6%). However, the logistic regression technique is of limited use in geographic areas aiming to begin a screening program, since the main predictors included in its equation are the percentage of times the diabetic had previously adhered and the result of the last screening. Nevertheless, this technique can be very useful and effective if the necessary data is available. The combined version 3 showed no overall improvement in comparison to version 2.

**Figure 8:**
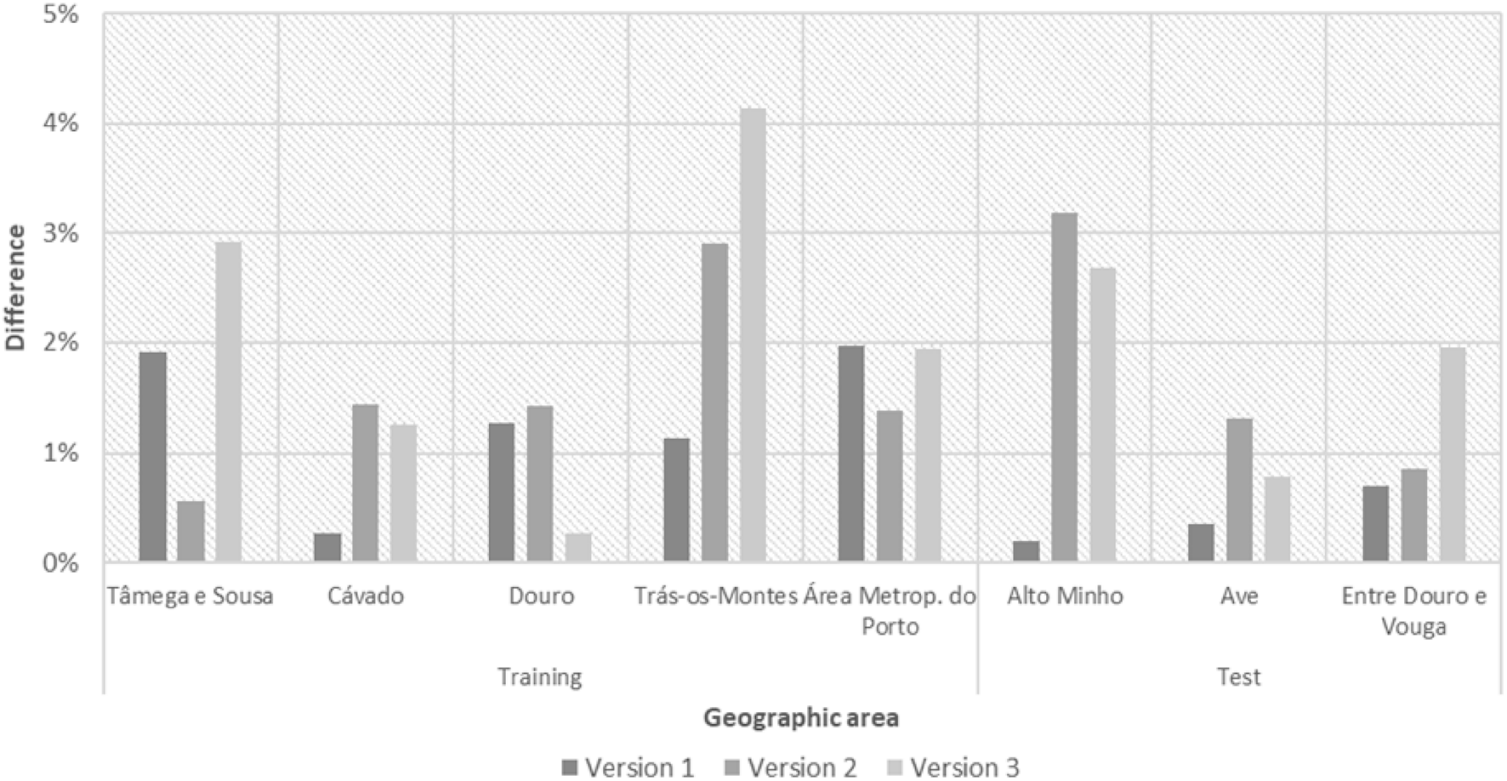
Simulation results versus real results in each of the ABM versions.

Figure 8 allows for direct comparation of the differences between the results obtained with each of the ABM versions and the real results, in all the geographic areas. As can be seen, most subregions follow the general trend, producing better results when using logistic regression only. However, in subregions where screening was started more recently (and therefore has fewer years of history), such as Douro and Tâmega e Sousa, the version that relies on fuzzy components or the combined version tend to have better results.

### Effect of different interventions on adherence rate

Since the region's adherence rate is lower than desired (80%), it will be necessary to develop public health interventions in this area. Therefore, in order to predict and compare the results of several possible interventions, simulations were carried out for different hypothetical scenarios. These are only first examples of the applications of our research (a prove of concept), and we plan to continue to improve our model so that it can be used to analyze a wider range of scenarios. All the simulations on this section were carried out using the ABM version with the logistic regression (version 1).

Scenario 1—intervention that allows increasing adherence of diabetics who tested positive in the previous screening to 95%

According to ARSN experts, it makes no sense for this group of diabetics to have lower adherence rates than those who had a negative result. Therefore, and based on the literature referred to previously^40^, the hypothesis was formulated that the existing data are biased due to a weak articulation between hospital services and primary health care, which leads to the sending of calls to diabetics followed in a hospital environment. In this way, this intervention would not actually consist of an increase in the real adherence rate, but rather an increase in the quality of data and the screening process.

Since only 4% of screening results are positive, a very significant impact on the overall adherence rate was not expected. In fact, the results of this simulation (in which the adherence rate of diabetics with a previous positive result was parameterized to 95% in all sub-regions) are in line with empirical knowledge, revealing that only the sub-regions with greater differences between the adherence of diabetics with previous negative and positive results show increases in the adherence rate (Table 2). Overall, the region's adherence rate would increase from 67.6 to 68.23%.

Scenario 2—Intervention that increases adherence by 5% of all diabetics who have already taken part in screening at least once.

**Table 2:** Scenarios results.

	Simulated adherence rate (%)		
	Without intervention	Scenario (1)	Difference
Scenario 1			
Alto Minho	74.30	74.94	0.64
Área Metropolitana do Porto	65.35	65.76	0.41
Ave	72.44	73.71	1.27
Cavado	66.75	67.27	0.52
Douro	68.13	68.19	0.06
Entre Douro e Vouga	66.95	66.98	0.03
Tâmega e Sousa	69.50	70.07	0.57
Trás-os-Montes	66.83	67.94	1.11
Scenario 2			
Alto Minho	74.30	77.14	2.84
Área Metropolitana do Porto	65.35	67.61	2.26
Ave	72.44	74.60	2.16
Cavado	66.75	69.06	2.31
Douro	68.13	70.53	2.40
Entre Douro e Vouga	66.95	68.75	1.80
Tâmega e Sousa	69.50	73.57	4.07
Trás-os-Montes	66.83	70.31	3.48
Scenario 3			
Alto Minho	74.30	74.32	0.01
Área Metropolitana do Porto	65.35	65.71	0.36
Ave	72.44	72.43	-0.01
Cavado	66.75	67.05	0.30
Douro	68.13	69.62	1.49
Entre Douro e Vouga	66.95	66.92	-0.03
Tâmega e Sousa	69.50	70.23	0.73
Trás-os-Montes	66.83	66.92	0.09

According to experts, this increase could be viable, taking advantage of the presence of diabetics at screening to provide them with a small training session on the disease and the importance of annual screenings.

Therefore, in this simulation, the adherence rate of all diabetics who have already taken part in screening was programed to be increased by 5%.

According to the results obtained, this intervention would lead to substantial increases in all sub-regions (Table 2) and an increase in the global adherence rate from 67.6 to 70.28%.

Scenario 3—Intervention that allows the adherence rate of younger diabetics, particularly students, to increase by 20%.

Although 20% is an ambitious increase, experts consider that it could be possible through information sessions in schools and with the collaboration of teachers. Therefore, in this simulation the adherence of diabetics under 25 years of age and students was programed to be increased by 20%.

Since the percentage of diabetics of school age is very small (only 5.2% of the total number of diabetics in the region) the impact of this measure is minimal in terms of increasing the overall adherence rate—from 67.6 to 67. 9%. The measure is a little more interesting in regions where the adherence rate of this group is extremely low, and/or where this age group is more significant (Table 2).

## Conclusions

This research aimed to demonstrate that it is possible to predict the rate of adherence to population-based screenings with a high level of abstraction using ABMs. More specifically, it intended: (i) to demonstrate that it is possible to develop an ABM that faithfully portrays the decision on whether to adhere to screening or not, using the intrinsic features of the agent and the screening program; (ii) to demonstrate that an ABM with the aforementioned characteristics can be used in contexts other than the one for which the data for its development were collected; (iii) to demonstrate the utility of combining ABM and fuzzy logic in models intended to simulate human behavior. To this end, three versions of an agent-based model were presented, differing in terms of the method used to infer the individual decision on whether to adhere to screening or not. For the first, we used a logistic regression equation, in the second logistic regression was replaced by three fuzzy logic components, and in the third a combination of the two methods was used. All three versions were calibrated and validated using real data from 271,867 calls for screening in the Northern Region Health Administration. The results obtained indicate that it is possible to predict the rate of adherence to screening for diabetic retinopathy using demographic and socioeconomic data for the target population, and information regarding the screening strategy. The use of the fuzzy components leads to a high level of abstraction from the real data and shows predictive capability in new contexts (the test set), which attests to the validity of the model for the study of this problem and its usefulness as a predictive tool for public health planning. In fact, the use of logistic regression (version 1) led to the best global result: a predicted adherence rate of 67.6%, a difference of only 1% in relation to the real value (66.6%). However, the logistic regression technique is of limited use in geographic areas aiming to begin a screening program, since the main predictors included in its equation are the percentage of times the diabetic had previously adhered and the result of the last screening. Nevertheless, this technique can be very useful and effective if the necessary data is available. The combined version 3 showed no overall improvement in comparison to version 2.

## Discussion

Since the 1990s, several simulation models focused on screening for diabetic retinopathy have been developed. However, despite the recognized importance of adherence to screening success, we only found one attempt to model the subjects' individual behavior^24^. This model incorporated the Health Belief Model (HBM) into a DES model through a combination of several factors (inputs) that influence screening adherence. The model used only artificial data, leading to theoretical results, which lack validation with real data. That research also stresses the difficulty of incorporating qualitative variables, such as those used by the HBM, in DES models, emphasizing the need for the use of another type of technique. The objective of our research was to overcome those limitations, proving that is possible to simulate screening adherence behavior using computer simulations, in particular agent-based models embedded with logistic regression or/and fuzzy logic components.

Regarding the logistic regression, we found that only three independent variables had predictive value: percentage of times the diabetic had previously adhered to the screening, the last screening result, and the number of times the diabetic was called for DR screening, which is in line with the literature^24^.

Has far as we know, our research is the first that aims to simulate adherence to RD screening using an ABM. However, ABM have been used quite successfully to model health behaviors, like alcohol use, diet, smoking.^25,26^. Techniques based on fuzzy logic have also been used for simulating human behavior with good results^30^. So, our results are in line with the literature, reinforcing the idea that these computational modeling techniques are very effective when it comes to human behavior, and are the first application on DR screenings. Moreover, we think we demonstrate the utility of the use of fuzzy logic embedded in an ABM that intend to simulate human behavior. As we did not find any research, in the health area, that combinates the two techniques we believe our results could be an important contribution to future research.

Despite the main focus of this article was proving that is possible to simulate adherence to population-based screenings through computational simulation models with a high level of abstraction, we also did some experiments to illustrate as such model can be used to support decisions in health planning, while analyzing the effectiveness of various interventions. For example, we studied the impact of promoting continuity in adherence to screening by providing the diabetic with a short training session on the day of the test, and of promoting adherence among younger diabetics through information sessions in schools. We are improving the model so that it can analyze a wider range of scenarios, the results of which we intend to present in our future research. We believe that the models developed can be of great importance when staging hypothetical interventions, enhancing the discovery of knowledge and when proposing measures to the public and private entities responsible for laws and decision-making. They can also facilitate the identification of groups/geographical locations where the problem of adherence to screening is particularly relevant and which factors have the greatest impact on the decision to adhere to the screening.

One limitation of this research is the fact that we did not have access to data from other locations with different screening strategies. In future, with the intention of improving the validation of our model, we intent to test our model with data from other geographic locations where both population characteristics and screening strategies differ substantially from those found in this training group. We also acknowledge that our approach relies on specific assumptions and data. To address data biases, we plan to test the model with data from other population-based screenings and gauge its ability to replicate the real-life adherence rates.

### Supplementary Information

Supplementary Information.

## Data Availability

The data that support the findings of this study are available from Portuguese North Region Health Administration, but restrictions apply to the availability of these data, which were used under license for the current study, and so are not publicly available. Data are however available from the authors upon reasonable request, addressed to the corresponding author, and with permission of the Portuguese North Region Health Administration.
