# Supplementary material for: The use of social simulation modelling to understand adherence to diabetic retinopathy screening programs
Source: Sci Rep. 2024 Feb 29;14:4963. doi: 10.1038/s41598-024-55517-4 (PMC10904866; doi:10.1038/s41598-024-55517-4)
Supplement: Supplementary file 1 — Supplementary Information. [file 41598_2024_55517_MOESM1_ESM.docx]

# **The use of social simulation modelling to understand adherence to diabetic retinopathy screening programs.**

Andreia Penso Pereira ^*, a^ , João Macedo ^b^, Ana Afonso ^c,^ Raul M. S. Laureano ^*, a, d^ , Fernando Buarque de Lima Neto ^e^

Afiliations:

^a^ Instituto Universitário de Lisboa (ISCTE-IUL), Information Sciences, Technologies and Architecture Research Center (ISTAR-IUL), Av. das Forças Armadas, 1649-026 Lisboa, Portugal

^b^ Universidade de Pernambuco (UPE), Escola Politécnica, Computer Engineering, (POLI/EComp), 50720-001 Recife, Brazil

^c^ Global Health and Tropical Medicine, GHTM, Associate Laboratory in Translation and Innovation Towards Global Health, LA-REAL, Instituto de Higiene e Medicina Tropical, IHMT, Universidade NOVA de Lisboa, UNL, Rua da Junqueira 100, 1349-008 Lisboa, Portugal^.^

^d^ Instituto Universitário de Lisboa (ISCTE-IUL), Business Research Unit (BRU-IUL), Av. das Forças Armadas, 1649-026 Lisboa, Portugal

^e^ Universidade de Pernambuco (UPE), Escola Politécnica, Computer Engineering (POLI/PPG-EC), Rua Benfica, 455 - Bloco 'C', 50720-001 Recife, Brazil

Authours emails:

Corresponding authour* Andreia Penso Pereira, [andreia_marisa_pereira@iscte-iul.pt](mailto:andreia_marisa_pereira@iscte-iul.pt)

Corresponding authour* Raul M. S. Laureano, [raul.laureano@iscte-iul.pt](mailto:raul.laureano@iscte-iul.pt)

Fernando Buarque de Lima Neto, [fbln@ecomp.poli.br](mailto:fbln@ecomp.poli.br)

João Macedo, [jssm@ecomp.poli.br](mailto:jssm@ecomp.poli.br)

Ana Afonso, [aafonso@ihmt.unl.pt](mailto:aafonso@ihmt.unl.pt)

* Corresponding author

**Supplementary Table S1 – State of the art of simulation models of DR screening**

|  | **Ref.** | **Authors** | **Year** | **Strengths and main outcomes** | **Limitations** |
| --- | --- | --- | --- | --- | --- |
| Cost-effectiveness of systematic DR screenings | 3 | J. C. Javitt, L. P. Aiello, Y. Chiang, F. L. Ferris, J. K. Canner and S. Greenfield | 1994 | Each diabetic is modelled individually, and the importance of DR population-based screening is highlighted. | The models include only a very limited set of risk factors, neglecting important aspects as adherence rate and sociodemographic factors; as some of the authors recognized, Monte Carlo simulation and Markov processes are not the most suitable techniques for modelling multiple individual characteristics. |
|  | 4 | A. J. Palmer, C. Weiss, P. P. Sendi, K. Neeser, A. Brandt, G. Singh, H. Wenzel and G. A. Spinas | 2000 | The rate of DR progression takes into consideration the duration of the diabetes, the blood glucose values, and insulin therapy, highlighting the importance of combining screening with proper control of diabetes. |  |
|  | 5 | B. A. Craig, D. G. Fryback, R. Klein and B. E. K. Klein | 1999 | The model allows for an assessment of the sensitivity of the results to each specific parameter and an estimation of joint uncertainty considering all model parameters. The model highlights the importance of DR population-based screening. |  |
|  | 6 | D. Vetrini, C. A. Kiire, I. P. Burgess, S. P. Harding, P. C. Kayange, K. Kalua, G. Msukwa, N. A. Beare and J. Madan | 2018 | The authors performed three “What If” scenarios with different adherence rates, revealing that the adherence rate has an important impact on the cost-effectiveness of the screening, since the highest costs are fixed and remain the same even when fewer screenings are performed. | No effort was made to model individual features; the underlying factors behind adherence rates were not considered, not even the impact of each of the proposed strategies (a fixed probability of adherence was parametrized for each set of simulations). |
| Screening alternatives and screening intervals | 7 | D. Maberley, H. Walker, A. Koushik and A. Cruess | 2003 | The authors modelled the cost-effectiveness of retinopathy screening by travelling retina specialists versus retinal photography with a portable digital camera, presenting a sensitivity analysis of different adherence rates or population coverage. It was demonstrated that highest percentages of adherence rates/ population coverage led to more cost-effective programs. |  |
|  | 8 | R. Davies and C. Canning | 1996 | The authors recommended annual screenings for diabetics without DR and 6-monthy screenings for diabetics with background DR. Another important finding in these studies was the important role of the population's compliance with screening. In fact, the authors report that the probability of a diabetic attending a screening when called significantly affects the screening results, influencing decisions such as the screening method, the professionals responsible for the initial test (in terms of sensitivity) and the intervals between screenings. | The individual features and state of health of diabetics are not considered in these models; the underlying factors behind adherence rates were not considered, (a fixed probability of adherence was parametrized for each set of simulations). DES has been found to be difficult to implement in situations involving human behavior, because entities in DES are not autonomous and capable of making independent decisions. Therefore, this is not the most suitable technique for representing complex proactive human |
|  | 9 | R. Davies, P. Sullivan and C. Canning | 1996 |  |  |
|  | 10 | R. Davies, S. Brailsford, P. Roderick, C. Canning and D. Crabbe | 2000 |  |  |
|  | 11 | R. Davies, P. Roderick, C. Canningt and S. Brailsford | 2002 |  |  |
|  | 12 | D. Chalk, M. Pitt, B. Vaidya and K. Stein | 2012 | The framework explicitly models each patient separately. Patient records included patient sex, type and duration of the diabetes, screening dates and last screening result. The proposed model allows for the possibility of non-attendance. The simulation predicts that screening people with type 2 diabetes, who have not yet developed DR, every two years does not increase the risk of vision loss and is cost-effective. | The model includes only a very limited set of risk and sociodemographic factors. The authors did not explore the impact of non-attendance on the costs and benefits of 2-year screening intervals. The underlying factors behind adherence rates were not considered. DES is not the most suitable technique to represent complex proactive human behavior. |
|  | 13 | S. Vijan, T. P. Hofer and R. A. Hayward | 2000 | One-way sensitive analyses were conducted on individual parameters to access their impact on the costs and effectiveness of the screening. The authors reported that annual retinal screening for all type 2 diabetic patients was not cost-effective and concluded that tailoring recommendations to individual circumstances may be preferable. | The individual features, state of health and adherence rate of diabetics were not considered in this model. Markov processes are not the most suitable technique for modelling multiple individual characteristics. |
|  | 14 | S. C. Brailsford, W. J. Gutjahr, M. S. Rauner and W. Zeppelzauer | 2007 | The study concluded that a 30-month screening interval was the most cost-effective option. In terms of simulation techniques, the authors propose a combined DES and ant colony optimization model. The effects of different screening strategies are simulated and then compared in terms of two objective functions: minimum incremental cost per year of sight saved, compared with no-screening, and maximum years of sight saved. | The individual features, state of health and adherence rate of diabetics were not considered in this model. The authors did not explore the impact of non-attendance. The underlying factors behind adherence rates were not addressed. |
|  | 15 | T. E. Day, N. Ravi, H. Xian and A. Brugh | 2013 | The authors present an Agent Based Model (ABM) supported by medical data abstracted from 535 patients’ records. Each agent is imbued with a data structure describing the demography and health status of the agent. The data abstraction was accomplished through probability density functions incorporated into the model. The variables included in the data structure are used as predictors for the DR progression, through a multivariate logistic regression model that provides the probability of transition from one state of the disease to another, individually, for each agent. The simulation results were validated against real-world data. In the continuation of this research the authors extended the model, integrating the previously developed ABM with a DES model that allows for the simulation of the path of a virtual cohort of diabetics in a screening and treatment clinic for DR. The results suggest that increasing the interval from 1 to 2 years for diabetic patients who have not yet developed DR is safe, while increasing the interval to 3 years increases the risk of vision loss. | The authors did not explore the impact of non-attendance. The underlying factors behind adherence rates were not considered. |
|  | 16 | T. E. Day, N. Ravi, H. Xian and A. Brugh | 2013 |  |  |
| The use of telemedicine in DR screening | 17 | N. Aoki, K. Dunn, T. Fukui, J. R. Beck, W. J. Schull and H. K. Li | 2004 | The main findings of the simulation were that the teleophthalmology system is more effective and less costly than the non-teleophthalmology system in the cost-effectiveness analysis for the reference case. | The individual features and adherence rate of diabetics were not considered in this model. Markov processes are not the most suitable technique for modelling multiple individual characteristics. The clinical effectiveness and economic value of telemedicine has not been clearly established. |
|  | 18 | J. D. Whited, S. K. Datta, L. M. Aiello, L. P. Aiello, J. D. Cavallerano, P. R. Conlin, M. B. Horton, R. A. Vigersky, R. K. Poropatich, P. Challa, A. Darkins and S.-E. Bursell | 2005 | The main findings of the simulation were that the teleophthalmology system is more effective and less costly than the non-teleophthalmology system. | The individual features and adherence rates of diabetics were not considered in this model. Monte Carlo simulation is not the most suitable technique for modelling multiple individual characteristics. |
|  | 19 | D. B. Rein, J. S. Wittenborn, X. Zhang, B. A. Allaire, M. S. Song, R. Klein and J. B. Saaddine | 2011 | The authors compare DR screening alternatives for diabetics with no or early DR, accounting for imperfect compliance with screening recommendations and the ability of eye tests to detect other common visual disorders in people with diabetes (glaucoma, aged-related macular degeneration, etc). There is a model validation process and a sensitivity analysis. This study concludes that biennial eye evaluation was the most cost-effective treatment option when the ability to detect other eye conditions was included in the model. Telemedicine was most cost-effective when other eye conditions were not considered. | The models include only a very limited set of risk and sociodemographic factors; Monte Carlo simulation and Markov processes are not the most suitable technique for modelling multiple individual characteristics. |
|  | 20 | E. Kirkizlar, N. Serban, J. A. Sisson, J. L. Swann, C. S. Barnes and M. D. Williams | 2013 | One important contribution of this study is the diversity of the population and geography, compared with earlier studies. The results concluded that telemedicine is cost-effective under most conditions and may increase screening rates. | Markov processes are not the most suitable technique for modelling multiple individual characteristics. |
|  | 21 | H. V. Nguyen, G. S. W. Tan, R. J. Tapp, S. Mital, D. S. W. Ting, H. T. Wong, C. S. Tan, A. Laude, E. S. Tai, N. C. Tan, E. A. Finkelstein, T. Y. Wong and E. L. Lamoureux | 2016 | Simulation results indicate that telemedicine-based DR screening saves costs and generates similar health outcomes | The individual features and adherence rates of diabetics were not considered in this model; Markov processes are not the most suitable technique for modelling multiple individual characteristics. |
|  | 22 | A. J. Ben, J. L. Neyeloff, C. F. Souza, A. P. O. Rosses, A. L. Araujo, A. Szortika, F. Locatelli, G. Carvalho and C. R. Neumann | 2020 |  | The individual features and adherence rate of diabetics were not considered in this model; Markov processes are not the most suitable technique for modelling multiple individual characteristics. The probability sensitivity analyses show a considerable amount of uncertainty in the model’s parameters |
| Human Behaviour and compliance with the screening | 23 | S. Brailsford and B. Schmidt | 2003 | This model uses a combination of several factors to study adherence to screening (number of times the patient has adhered to previous screenings, perception of their general health status, current stage of the DR, information and anxiety about the DR, and educational qualifications). Each patient is an individual entity in the model, with their own characteristics. | The probability of participation in the screening was calculated simply as a binary variable and the model uses only artificial data, meaning that the results of this model are theoretical artefacts which need to be validated with real data. Qualitative variables are difficult to incorporate into DES models. |

**Supplementary Information S2-** **Fuzzy IF-THEN rules**

1. **Access barriers**

Variables:

B1 - age.

“Difficult access due to age” is defined by the linear function that passes through the points [0, 1], [100, 0]. “Easy access” is defined by the linear function that passes through the points [0, 0] and [100, 1].

B2 - income.

"Difficult access due to income" is defined by the normal distribution of the mean 50,000 euros/year and standard deviation 17,000 euros/year. The classification "easy access due to income" corresponds to the maximum of two normal distributions with averages of 0 and 100,000 euros/ year respectively and standard deviations of 17,000 euros/year.

B3 - screening location

“Difficult to access due to screening location” is defined by the linear function that passes through the points [0, 0], [100, 1]. “Easy access due to screening location” is defined by the linear function that passes through the points [0, 1] and [100, 0].

B4 - degree of urbanization of the place of residence.

The “difficult access due to the degree of urbanization” is defined by the normal distribution of mean 0.3 and standard deviation 0.1. The classification “easy access due to the degree of urbanization” corresponds to the maximum of two normal distributions with means 0 and 0.5 respectively and standard deviations 0.1.

Rules:

R1: IF (B1 is high_B1 AND B2 is high_B2 AND B3 is high_B3 And B4 is high_B4), THEN it is likely that I will attend_screening.

R2: IF (B1 is high_B1 AND B2 is low_B2 AND B3 is high_B3 And B4 is high_B4), THEN it is likely that I will attend_screening.

R3: IF (B1 is high_B1 AND B2 is high _B2 AND B3 is low_B3 And B4 is high_B4), THEN it is likely that I will attend_screening.

R4: IF (B1 is high_B1 AND B2 is high _B2 AND B3 is low_B3 And B4 is high_B4), THEN it is likely that I will attend_screening.

R5: IF (B1 is high_B1 AND B2 is high _B2 AND B3 is high_B3 And B4 is low _B4), THEN it is likely that I will attend_screening.

R6: IF (B1 is low_B1 AND B2 is low_B2 AND B3 is high_B3 And B4 is high_B4), THEN it is likely that I will attend_screening.

R7: IF (B1 is low_B1 AND B2 is high_B2 AND B3 is low_B3 And B4 is high_B4), THEN it is likely that I will attend_screening.

R8: IF (B1 is low_B1 AND B2 is high_B2 AND B3 is high_B3 And B4 is low_B4), THEN it is likely that I will attend_screening.

R9: IF (B1 is high_B1 AND B2 is low_B2 AND B3 is low_B3 And B4 is high_B4), THEN it is likely that I will attend_screening.

R10: IF (B1 is high_B1 AND B2 is low_B2 AND B3 is high_B3 And B4 is low _B4), THEN it is likely that I will attend_screening.

R11: IF (B1 is high_B1 AND B2 is high_B2 AND B3 is low_B3 And B4 is low_B4), THEN it is likely that I will attend_screening.

R12: IF (B1 is high_B1 AND B2 is low_B2 AND B3 is low_B3 And B4 is low_B4), THEN it is unlikely that I will attend_screening.

R13: IF (B1 is low_B1 AND B2 is high_B2 AND B3 is low_B3 And B4 is low_B4), THEN it is unlikely that I will attend_screening.

R14: IF (B1 is low_B1 AND B2 is low_B2 AND B3 is high_B3 And B4 is low_B4), THEN it is unlikely that I will attend_screening.

R15: IF (B1 is low_B1 AND B2 is low_B2 AND B3 is low_B3 And B4 is high_B4), THEN it is unlikely that I will attend_screening.

R16: IF (B1 is low_B1 AND B2 is low_B2 AND B3 is low_B3 And B4 is low_B4), THEN it is unlikely that I will attend_screening.

1. **Knowledge of the disease component**

Variables:

C1 - age.

"High knowledge level due to age" is defined by a normal distribution of the mean 65 years and standard deviation 30. "Low knowledge level due to age" corresponds to the maximum of two normal distributions with means 18 and 100 years respectively and deviation pattern 30.

C2 - educational qualifications.

“High knowledge level due to educational qualifications” is defined by a linear function that passes through the points [0, 0] and [100, 1]. “Low knowledge level due to educational qualifications” is defined by a linear function that passes through the points [0, 1] and [100, 0].

C3 - percentage of times the agent previously adhered to screening.

“High knowledge level due to prior adhesion” is defined by a linear function that passes through the points [0, 0] and [100, 1]. “Low knowledge level due to prior adhesion” is defined by a linear function that passes through the points [0, 1] and [100, 0].

Rules:

R1: IF (C1 is high_C1 AND C2 is high_C2 AND C3 is high_C3), THEN it is likely that I will attend_screening.

R2: IF (C1 is high_C1 AND C2 is low_C2 AND C3 is high_C3), THEN it is likely that I will attend_screening.

R3: IF (C1 is high_C1 AND C2 is high_C2 AND C3 is low_C3), THEN it is likely that I will attend_screening.

R4: IF (C1 is low_C1 AND C2 is high_C2 AND C3 is high_C3), THEN it is likely that I will attend_screening.

R5: IF (C1 is high_C1 AND C2 is low_C2 AND C3 is low_C3), THEN it is unlikely that I will attend_screening.

R6: IF (C1 is low_C1 AND C2 is high_C2 AND C3 is low_C3), THEN it is unlikely that I will attend_screening.

R7: IF (C1 is low_C1 AND C2 is low_C2 AND C3 is high_C3), THEN it is unlikely that I will attend_screening.

R8: IF (C1 is low_C1 AND C2 is low_C2 AND C3 is low_C3), THEN it is unlikely that I will attend_screening.

1. **Quality/strategy of the screening program**

Variables:

E1 - sending reminders.

“High quality, considering sending reminders” is defined by a linear function that passes through the points [0, 0] and [100, 1]. “Low quality, considering sending reminders” is defined by a linear function that passes through the points [0, 1] and [100, 0].

E2 - waiting time at the time of screening (in minutes).

“High quality, considering the waiting time” is defined by a linear function that passes through the points [0, 1] and [500, 0]. “Low quality, considering the waiting time” is defined by a linear function that passes through the points [0, 0] and [500, 1].

E3 - time (in weeks) between sending the call notice and the date of the screening.

. "High quality, considering advance notification of the call" is defined by a normal distribution of mean 4 and standard deviation 2. "Low quality, considering advance notification of the call" corresponds to the maximum of two normal distributions with means 0 and 8 respectively and standard deviations 2.

Rules:

R1: IF (E1 is high_E1 AND E2 is high_E2 AND E3 is high_E3), THEN it is likely that I will attend_screening.

R2: IF (E1 is high_E1 AND E2 is low_E2 AND E3 is high_E3), THEN it is likely that I will attend_screening.

R3: IF (E1 is high_E1 AND E2 is high_E2 AND E3 is low_E3), THEN it is likely that I will attend_screening.

R4: IF (E1 is low_E1 AND E2 is high_E2 AND E3 is high_E3), THEN it is likely that I will attend_screening.

R5: IF (E1 is high_E1 AND E2 is low_E2 AND E3 is low_E3), THEN it is unlikely that I will attend_screening.

R6: IF (E1 is low_E1 AND E2 is high_E2 AND E3 is low_E3), THEN it is unlikely that I will attend_screening.

R7: IF (E1 is low_E1 AND E2 is low_E2 AND E3 is high_E3), THEN it is unlikely that I will attend_screening.

R8: IF (E1 is low_E1 AND E2 is low_E2 AND E3 is low_E3), THEN it is unlikely that I will attend_screening.

**Supplementary Table S3 – TwoStep Cluster Analysis**

| Model Summary  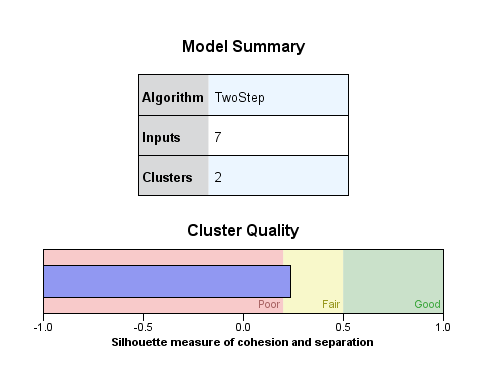 | 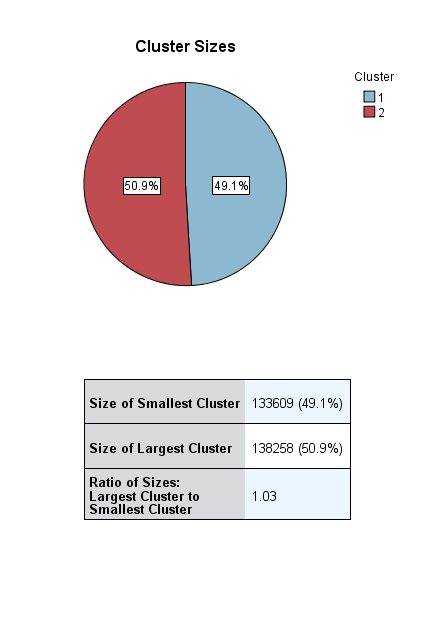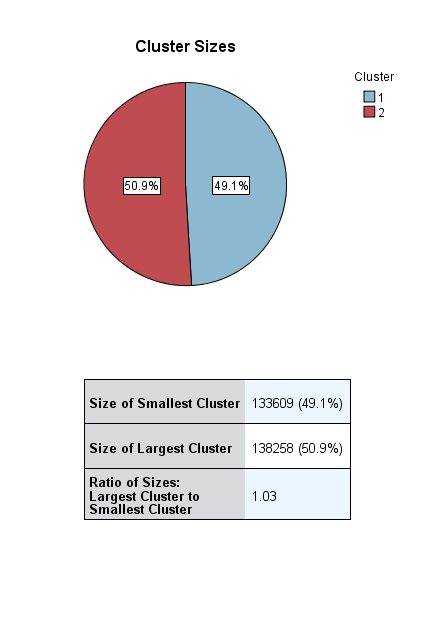Cluster Sizes | Predictor importance  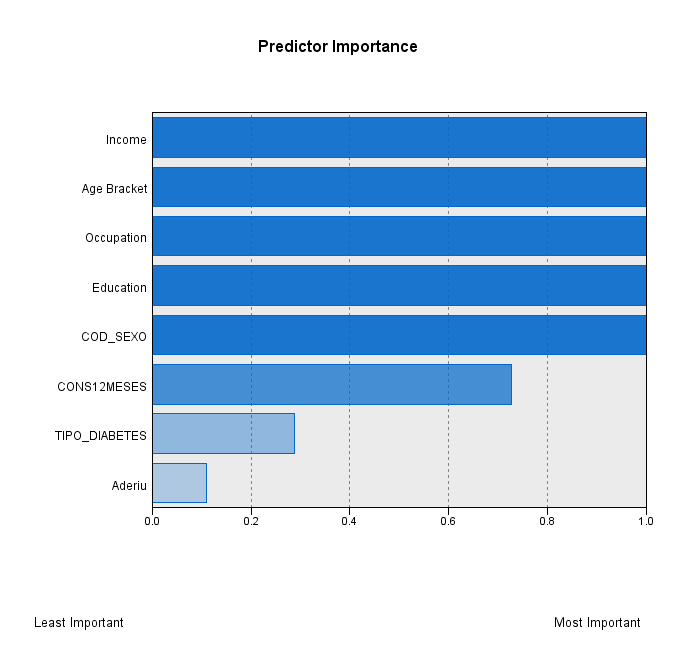 |
| --- | --- | --- |

**Supplementary Table S4 –Cluster Analysis – Crosstabulation, Chi-saquare and V’Cramer tests**

|  | Crosstabulation | Chi-saquare | V’Cramer |
| --- | --- | --- | --- |
| Age Bracket | \|  \| \| \| \| TwoStep Cluster Number \| \| \| \| Total \| \| \| --- \| --- \| --- \| --- \| --- \| --- \| --- \| --- \| --- \| --- \| \| 1 \| \| 2 \| \| \|  \| 18-54 \| \| Count \| \| 0 \| \| 1796 \| \| 1796 \| \| \| Expected Count \| \| 882.6 \| \| 913.4 \| \| 1796.0 \| \| \| % within Age Bracket \| \| 0.0% \| \| 100.0% \| \| 100.0% \| \| \| 54-64 \| \| Count \| \| 0 \| \| 14463 \| \| 14463 \| \| \| Expected Count \| \| 7107.8 \| \| 7355.2 \| \| 14463.0 \| \| \| % within Age Bracket \| \| 0.0% \| \| 100.0% \| \| 100.0% \| \| \| 64-74 \| \| Count \| \| 54 \| \| 89494 \| \| 89548 \| \| \| Expected Count \| \| 44008.4 \| \| 45539.6 \| \| 89548.0 \| \| \| % within Age Bracket \| \| 0.1% \| \| 99.9% \| \| 100.0% \| \| \| >74 \| \| Count \| \| 133555 \| \| 32505 \| \| 166060 \| \| \| Expected Count \| \| 81610.2 \| \| 84449.8 \| \| 166060.0 \| \| \| % within Age Bracket \| \| 80.4% \| \| 19.6% \| \| 100.0% \| \| \| Total \| \| Count \| \| \| 133609 \| \| 138258 \| \| 271867 \| \| \| Expected Count \| \| \| 133609.0 \| \| 138258.0 \| \| 271867.0 \| \| \| % within Age Bracket \| \| \| 49.1% \| \| 50.9% \| \| 100.0% \| \| | \|  \| Value \| df \| Asymptotic Significance  (2-sided) \| \| --- \| --- \| --- \| --- \| \| Pearson  Chi-Square \| 167050.92^a^ \| 3 \| .000 \| \| Likelihood  Ratio \| 211685.04 \| 3 \| .000 \| \| N of Valid  Cases \| 271867 \|  \|  \| \|  \| \| \| \| | \|  \| \| Value \| Approximate Significance \| \| --- \| --- \| --- \| --- \| \|  \| Cramer's V \| .784 \| .000 \| \| N of Valid Cases \| \| 271867 \|  \| |
| Gender | \|  \| \| \| TwoStep Cluster Number \| \| Total \| \| --- \| --- \| --- \| --- \| --- \| --- \| \| 1 \| 2 \| \|  \| M \| Count \| 54535 \| 80139 \| 134674 \| \| Expected Count \| 66185.5 \| 68488.5 \| 134674.0 \| \| % within COD_SEXO \| 40.5% \| 59.5% \| 100.0% \| \| W \| Count \| 79074 \| 58119 \| 137193 \| \| Expected Count \| 67423.5 \| 69769.5 \| 137193.0 \| \| % within COD_SEXO \| 57.6% \| 42.4% \| 100.0% \| \| Total \| \| Count \| 133609 \| 138258 \| 271867 \| \| Expected Count \| 133609.0 \| 138258.0 \| 271867.0 \| \| % within COD_SEXO \| 49.1% \| 50.9% \| 100.0% \| | \|  \| Value \| df \| Asymptotic Significance (2-sided) \| \| --- \| --- \| --- \| --- \| \| Pearson  Chi-Square \| 7991.32^a^ \| 1 \| .000 \| \| Likelihood  Ratio \| 8031.30 \| 1 \| .000 \| \| N of Valid  Cases \| 271867 \|  \|  \| | \|  \| \| Value \| Approximate Significance \| \| --- \| --- \| --- \| --- \| \|  \| Cramer's V \| .171 \| .000 \| \| N of Valid Cases \| \| 271867 \|  \| |
| Education | \|  \| \| \| TwoStep Cluster Number \| \| Total \| \| --- \| --- \| --- \| --- \| --- \| --- \| \| 1 \| 2 \| \|  \| KS2 \| Count \| 32085 \| 17290 \| 49375 \| \| Expected Count \| 24265.3 \| 25109.7 \| 49375.0 \| \| % within Education \| 65.0% \| 35.0% \| 100.0% \| \| KS3 \| Count \| 68722 \| 89917 \| 158639 \| \| Expected Count \| 77963.1 \| 80675.9 \| 158639.0 \| \| % within Education \| 43.3% \| 56.7% \| 100.0% \| \| KS4 \| Count \| 17312 \| 16410 \| 33722 \| \| Expected Count \| 16572.7 \| 17149.3 \| 33722.0 \| \| % within Education \| 51.3% \| 48.7% \| 100.0% \| \| KS5 or more \| Count \| 15490 \| 14641 \| 30131 \| \| Expected Count \| 14807.9 \| 15323.1 \| 30131.0 \| \| % within Education \| 51.4% \| 48.6% \| 100.0% \| \| Total \| \| Count \| 133609 \| 138258 \| 271867 \| \| Expected Count \| 133609.0 \| 138258.0 \| 271867.0 \| \| % within Education \| 49.1% \| 50.9% \| 100.0% \| | \|  \| Value \| df \| Asymptotic Significance  (2-sided) \| \| --- \| --- \| --- \| --- \| \| Pearson  Chi-Square \| 7235.685^a^ \| 3 \| .000 \| \| Likelihood  Ratio \| 7310.902 \| 3 \| .000 \| \| N of Valid  Cases \| 271867 \|  \|  \| | \|  \| \| Value \| Approximate Significance \| \| --- \| --- \| --- \| --- \| \|  \| Cramer's V \| .163 \| .000 \| \| N of Valid Cases \| \| 271867 \|  \| |
| Income (euros) | \|  \| \| \| TwoStep Cluster Number \| \| Total \| \| --- \| --- \| --- \| --- \| --- \| --- \| \| 1 \| 2 \| \|  \| <8511 \| Count \| 1898 \| 1657 \| 3555 \| \| Expected Count \| 1747.1 \| 1807.9 \| 3555.0 \| \| % within Income \| 53.4% \| 46.6% \| 100.0% \| \| 8511-9811 \| Count \| 54844 \| 41870 \| 96714 \| \| Expected Count \| 47530.1 \| 49183.9 \| 96714.0 \| \| % within Income \| 56.7% \| 43.3% \| 100.0% \| \| 911-11167 \| Count \| 68924 \| 86862 \| 155786 \| \| Expected Count \| 76561.0 \| 79225.0 \| 155786.0 \| \| % within Income \| 44.2% \| 55.8% \| 100.0% \| \| 11167-12649 \| Count \| 3960 \| 4626 \| 8586 \| \| Expected Count \| 4219.6 \| 4366.4 \| 8586.0 \| \| % within Income \| 46.1% \| 53.9% \| 100.0% \| \| 12649-17400 \| Count \| 3178 \| 2437 \| 5615 \| \| Expected Count \| 2759.5 \| 2855.5 \| 5615.0 \| \| % within Income \| 56.6% \| 43.4% \| 100.0% \| \| >17400 \| Count \| 805 \| 806 \| 1604 \| \| Expected Count \| 788.3 \| 815.7 \| 1604.0 \| \| % within Income \| 50.2% \| 49.8% \| 100.0% \| \| Total \| \| Count \| 133609 \| 138258 \| 271867 \| \| Expected Count \| 133609.0 \| 138258.0 \| 271867.0 \| \| % within Income \| 49.1% \| 50.9% \| 100.0% \| | \|  \| Value \| df \| Asymptotic Significance  (2-sided) \| \| --- \| --- \| --- \| --- \| \| Pearson  Chi-Square \| 3900.36^a^ \| 6 \| .000 \| \| Likelihood  Ratio \| 3912.12 \| 6 \| .000 \| \| N of Valid  Cases \| 271867 \|  \|  \| | \|  \| \| Value \| Approximate Significance \| \| --- \| --- \| --- \| --- \| \|  \| Cramer's V \| .120 \| .000 \| \| N of Valid Cases \| \| 271867 \|  \| |
| Occupation | \|  \| \| \| TwoStep Cluster Number \| \| Total \| \| --- \| --- \| --- \| --- \| --- \| --- \| \| 1 \| 2 \| \|  \| Active \| Count \| 16865 \| 94623 \| 111488 \| \| Expected Count \| 54790.8 \| 56697.2 \| 111488.0 \| \| % within Occupation \| 15.1% \| 84.9% \| 100.0% \| \| Inactive \| Count \| 4064 \| 28414 \| 32478 \| \| Expected Count \| 15961.3 \| 16516.7 \| 32478.0 \| \| % within Occupation \| 12.5% \| 87.5% \| 100.0% \| \| Retired \| Count \| 112390 \| 13286 \| 125676 \| \| Expected Count \| 61763.5 \| 63912.5 \| 125676.0 \| \| % within Occupation \| 89.4% \| 10.6% \| 100.0% \| \| Student \| Count \| 2 \| 1008 \| 1010 \| \| Expected Count \| 496.4 \| 513.6 \| 1010.0 \| \| % within Occupation \| 0.2% \| 99.8% \| 100.0% \| \| Unknow \| Count \| 288 \| 927 \| 1215 \| \| Expected Count \| 597.1 \| 617.9 \| 1215.0 \| \| % within Occupation \| 23.7% \| 76.3% \| 100.0% \| \| Total \| \| Count \| 133609 \| 138258 \| 271867 \| \| Expected Count \| 133609.0 \| 138258.0 \| 271867.0 \| \| % within Occupation \| 49.1% \| 50.9% \| 100.0% \| | \|  \| Value \| df \| Asymptotic Significance  (2-sided) \| \| --- \| --- \| --- \| --- \| \| Pearson  Chi-Square \| 151942.13^a^ \| 4 \| .000 \| \| Likelihood  Ratio \| 171391.65 \| 4 \| .000 \| \| N of Valid  Cases \| 271867 \|  \|  \| | \|  \| \| Value \| Approximate Significance \| \| --- \| --- \| --- \| --- \| \|  \| Cramer's V \| .748 \| .000 \| \| N of Valid Cases \| \| 271867 \|  \| |
| Consultation in the last 12 months | \|  \| \| \| TwoStep Cluster Number \| \| Total \| \| --- \| --- \| --- \| --- \| --- \| --- \| \| 1 \| 2 \| \|  \| No \| Count \| 1695 \| 4233 \| 5928 \| \| Expected Count \| 2913.3 \| 3014.7 \| 5928.0 \| \| % within CONS12MESES \| 28.6% \| 71.4% \| 100.0% \| \| Yes \| Count \| 131914 \| 134025 \| 265939 \| \| Expected Count \| 130695.7 \| 135243.3 \| 265939.0 \| \| % within CONS12MESES \| 49.6% \| 50.4% \| 100.0% \| \| Total \| \| Count \| 133609 \| 138258 \| 271867 \| \| Expected Count \| 133609.0 \| 138258.0 \| 271867.0 \| \| % within CONS12MESES \| 49.1% \| 50.9% \| 100.0% \| | \|  \| Value \| df \| Asymptotic Significance  (2-sided) \| \| --- \| --- \| --- \| --- \| \| Pearson  Chi-Square \| 1024.171^a^ \| 1 \| .000 \| \| Likelihood  Ratio \| 1059.768 \| 1 \| .000 \| \| N of Valid  Cases \| 271867 \|  \|  \| | \|  \| \| Value \| Approximate Significance \| \| --- \| --- \| --- \| --- \| \|  \| Cramer's V \| .061 \| .000 \| \| N of Valid Cases \| \| 271867 \|  \| |
| Type of diabetes | \|  \| \| \| TwoStep Cluster Number \| \| Total \| \| --- \| --- \| --- \| --- \| --- \| --- \| \| 1 \| 2 \| \|  \| Tipo I \| Count \| 10721 \| 14163 \| 24884 \| \| Expected Count \| 12229.2 \| 12654.8 \| 24884.0 \| \| % within TIPO_DIABETES \| 43.1% \| 56.9% \| 100.0% \| \| Tipo II \| Count \| 122888 \| 124095 \| 246983 \| \| Expected Count \| 121379.8 \| 125603.2 \| 246983.0 \| \| % within TIPO_DIABETES \| 49.8% \| 50.2% \| 100.0% \| \| Total \| \| Count \| 133609 \| 138258 \| 271867 \| \| Expected Count \| 133609.0 \| 138258.0 \| 271867.0 \| \| % within TIPO_DIABETES \| 49.1% \| 50.9% \| 100.0% \| | \|  \| Value \| df \| Asymptotic Significance (2-sided) \| \| --- \| --- \| --- \| --- \| \| Pearson  Chi-Square \| 402.621^a^ \| 1 \| .000 \| \| Likelihood  Ratio \| 404.029 \| 1 \| .000 \| \| N of Valid  Cases \| 271867 \|  \|  \| | \|  \| \| Value \| Approximate Significance \| \| --- \| --- \| --- \| --- \| \|  \| Cramer's V \| .038 \| .000 \| \| N of Valid Cases \| \| 271867 \|  \| |

**Supplementary Table S5 - Distribution of sociodemographic, health service utilization,**

**health status and screening features by screening adherence**

| **Category** | **Variable** | | **Screening adherence** | | | | | | **Test results** |
| --- | --- | --- | --- | --- | --- | --- | --- | --- | --- |
|  |  | | **No** | | | **Yes** | | |  |
| **Sociodemographic** | **Age** | |  | | |  | | |  |
|  | 18-39 39-54 54-64 64-74 >74 | | 53.60% 37.25% 29.79% 28.71% 42.54% | | | 46.40% 62.75% 70.21% 71.29% 57.46% | | | X2=861.,57 (p_value=0.00) Cramer's V=0.14 |
|  | **Gender** | |  | | |  | | |  |
|  | M W | | 32.87% 36.92% | | | 67.13% 63.08% | | | X2=83.41 (p_value=0.00) Cramer's V=0.03 |
|  | **Degree of urbanization of the area of residence** | |  | | |  | | |  |
|  | 0 1 2 5 | | 34.33% 39.07% 34.08% 34.27% | | | 65.67% 60.93% 65.92% 65.73% | | | X2=74.94 (p_value=0.00) Cramer's V=0.06 |
|  | **Professional status** | |  | | |  | | |  |
|  | Active Unknown Student Not active Retired | | 32.03% 41.09% 55.83% 33.03% 37.78% | | | 67.97% 58.91% 44.17% 66.97% 62.22% | | | X2=181.23 (p_value=0.00) Cramer's V=0.06 |
|  | **Existence of telephone contact for sending reminders** | |  | | |  | | |  |
|  | N Y | | 45.18% 33.12% | | | 54.82% 66.88% | | | X2=383.60 (p_value=0.00) Cramer's V=0.09 |
|  | **Income (median)** | |  | | |  | | |  |
|  | Unknown <8511 8511-9811 9811-11167 11167-12649 12649-17400 >17400 | | 38.57% 36.12% 33.98% 31.76% 34.16% 37.16% 38.18% | | | 61.43% 63.88% 66.02% 68.24% 65.84% 62.84% 61.82% | | | X2=97.67 (p_value=0.00) Cramer's V=0.05 |
|  | **Education** | | |  | | |  | |  |
|  | KS2  KS3  KS4  KS5  College degree | 38.57% 35.45% 33.54% 36.33% 38.18% | | | 61.43% 64.55% 66.46% 61.82% 63.67% | | | X2=60.02 (p_value=0.00) Cramer's V=0.04 | |
| **Relationship with health services** | **Type of Health Unit** | | |  | | |  | |  |
|  | UCSP USF A USF B | 38.29% 37.57% 33.06% | | | 61.71% 62.43% 66.94% | | | | X2=114.38 (p_value=0.00) Cramer's V=0.04 |
|  | **Family doctor** |  | | |  | | | |  |
|  | N Y | 45.26% 34.93% | | | 54.74% 65.07% | | | | X2=114.38 (p_value=0.00) Cramer's V=0.04 |
|  | **Exemption from charges for services** |  | | |  | | | |  |
|  | Not exempt Insufficient income Exempt for another reason | 33.57% 35.59% 38.70% | | | 66.43% 64.41% 61.30% | | | | X2=35.42 (p_value=0.00) Cramer's V=0.03 |
|  | **Number of consultations at the Primary Care Unit in the last 12 months** | | |  | | |  | |  |
|  | 0 1 2-3 4-6 7-9 >=10 | 71.59% 60.30% 34.83% 33.69% 32.47% 36.55% | | | 28.41% 39.70% 65.17% 66.32% 67.53% 63.45% | | | X2=501.62 (p_value=0.00) Cramer's V=0.10 | |
| **Health status** | **Type of diabetes (I or II)** |  | | |  | | |  | |
|  | Type I Type II | 44.34% 34.12% | | | 55.66% 65.83% | | | X2=8.89 (p_value=0.00) Cramer's V=0.01 | |
|  | **Body Mass Index (BMI)** |  | | |  | | |  | |
|  | NA <18.5 18.5-24.9 25-30 >=30 | 37.99% 38.46% 33.05% 28.19% 29.26% | | | 62.01% 61.54% 66.95% 71.81% 70.74% | | | X2=362.91 (p_value=0.00) Cramer's V=0.09 | |
|  | **Blood glucose levels (HBA1C)** |  | | |  | | |  | |
|  | NA <8 >=8 | 37.97% 28.54% 34.90% | | | 62.03% 71.46% 65.10% | | | X2=373.87 (p_value=0.00) Cramer's V=0.09 | |
| **DR Screening** | **Days elapsed between calls** |  | | |  | | |  | |
|  | NA <365 365-455 455-545 545-635 >=635 | 32.08% 36.94% 34.92% 33.51% 33.82% 37.16% | | | 67.92% 63.06% 65.08% 66.49% 66.18% 62.84% | | | X2=64.41 (p_value=0.00) Cramer's V=0.04 | |
|  | **Month of call for screening** |  | | |  | | |  | |
|  | 01 02 03 04 05 06 07 08 09 10 11 12 | 34.84% 34.73% 31.34% 31.98% 34.45% 43.18% 33.67% 36.23% 31.17% 33.46% 36.36% 47.20% | | | 65.16% 65.27% 68.66% 68.02% 65.55% 56.82% 66.33% 63.77% 68.83% 66.54% 63.64% 52.80% | | | X2=277.22 (p_value=0.00) Cramer's V=0.08 | |
|  | **Number of times the diabetic was called** |  | | |  | | |  | |
|  | 1 2 3 4 5 6 | 32.49% 37.63% 36.46% 31.94% 23.32% 21.43% | | | 67.51% 62.37% 63.54% 68.06% 76.68% 78.57% | | | X2=184.80 (p_value=0.00) Cramer's V=0.06 | |
|  | **Last screening result** |  | | |  | | |  | |
|  | NA Did not attend Negative Inconclusive Positive | 31.09% 60.71% 20.62% 33.08% 38.80% | | | 68.91% 39.29% 79.38% 66.92% 61.20% | | | X2=6318.43 (p_value=0.00) Cramer's V=0.37 | |
|  | **Percentage of times the diabetic attended previous screenings** |  | | |  | | |  | |
|  | NA 0% 25%-50% 50%-75% >=75% | 32.08% 68.11% 56.80% 36.67% 19.17% | | | 67.92% 31.90% 43.20% 63.33% 80.83% | | | X2=7095.41 (p_value=0.00) Cramer's V=0.39 | |
